# Influence of Aldehyde Dehydrogenase Inhibition on Stemness of Endometrial Cancer Stem Cells

**DOI:** 10.3390/cancers16112031

**Published:** 2024-05-27

**Authors:** Beatriz Serambeque, Catarina Mestre, Gabriela Correia-Barros, Ricardo Teixo, Carlos Miguel Marto, Ana Cristina Gonçalves, Francisco Caramelo, Isabel Silva, Artur Paiva, Hans C. Beck, Ana Sofia Carvalho, Maria Filomena Botelho, Maria João Carvalho, Rune Matthiesen, Mafalda Laranjo

**Affiliations:** 1 Univ Coimbra, Coimbra Institute for Clinical and Biomedical Research (iCBR) Area of Environment Genetics and Oncobiology (CIMAGO), Institute of Biophysics, Faculty of Medicine, 3000-548 Coimbra, Portugal; uc48103@uc.pt (C.M.); uc2019169084@student.uc.pt (G.C.-B.); uc2008114703@student.uc.pt (R.T.); cmiguel.marto@uc.pt (C.M.M.); mfbotelho@fmed.uc.pt (M.F.B.); mjcarvalho@fmed.uc.pt (M.J.C.); 2 Univ Coimbra, Center for Innovative Biomedicine and Biotechnology (CIBB), 3000-548 Coimbra, Portugal; acgoncalves@fmed.uc.pt (A.C.G.); fcaramelo@fmed.uc.pt (F.C.); artur.paiva@chuc.min-saude.pt (A.P.); 3 Univ Coimbra, Institute of Experimental Pathology, Faculty of Medicine, 3000-548 Coimbra, Portugal; 4 Clinical Academic Centre of Coimbra (CACC), 3004-561 Coimbra, Portugal; 5 Univ Coimbra, Coimbra Institute for Clinical and Biomedical Research (iCBR) Area of Environment Genetics and Oncobiology (CIMAGO), Laboratory of Oncobiology and Hematology (LOH) and University Clinics of Hematology and Oncology, Faculty of Medicine, 3000-548 Coimbra, Portugal; 6 Univ Coimbra, Coimbra Institute for Clinical and Biomedical Research (iCBR) Area of Environment Genetics and Oncobiology (CIMAGO) and Laboratory of Biostatistics and Medical Informatics (LBIM), Faculty of Medicine, 3004-531 Coimbra, Portugal; 7 Cytometry Operational Management Unit, Clinical Pathology Department, Unidade de Saúde Local de Coimbra, 3004-561 Coimbra, Portugal; 14546@ulscoimbra.min-saude.pt; 8 Polytechnic Institute of Coimbra, Coimbra Health School, Laboratory Biomedical Sciences, 3045-043 Coimbra, Portugal; 9 Department of Clinical Biochemistry, Odense University Hospital, 5000 Odense, Denmark; hcbeck@health.sdu.dk; 10 iNOVA4Health, NOVA Medical School (NMS), Faculdade de Ciências Médicas (FCM), Universidade Nova de Lisboa, 1150-082 Lisboa, Portugal; ana.carvalho@nms.unl.pt (A.S.C.); rune.matthiesen@nms.unl.pt (R.M.); 11 Univ Coimbra, Universitary Clinic of Gynecology, Faculty of Medicine, 3004-561 Coimbra, Portugal; 12 Gynecology Service, Department of Gynecology, Obstetrics, Reproduction and Neonatology, Unidade Local de Saúde de Coimbra, 3004-561 Coimbra, Portugal

**Keywords:** aldehyde dehydrogenase, ALDH inhibition, cancer stem cells, endometrial neoplasms, N,N-diethylaminobenzaldehyde, proteomics, stemness

## Abstract

**Simple Summary:**

Cancer stem cells (CSCs) are responsible for tumour initiation, growth, dissemination, and resistance to therapy. In this study, we intended to evaluate the effect of inhibiting aldehyde dehydrogenase (ALDH), an enzyme found in endometrial CSCs, using N,N-diethylaminobenzaldehyde (DEAB). Our findings suggest that ALDH blockage mediated by DEAB reduces enzyme activity and expression and can modulate CSC phenotype and behaviour. Additionally, DEAB modulates the expression of certain proteins, namely, ALDH18A1, SdhA, and UBAP2L, identified in endometrial cancer, which might be promising prognostic and therapeutic targets. ALDH inhibition could be a potential strategy to prevent endometrial CSCs from growing and spreading.

**Abstract:**

Endometrial cancer is one of the most common gynaecological malignancies. Although often diagnosed at an early stage, there is a subset of patients with recurrent and metastatic disease for whom current treatments are not effective. Cancer stem cells (CSCs) play a pivotal role in triggering tumorigenesis, disease progression, recurrence, and metastasis, as high aldehyde dehydrogenase (ALDH) activity is associated with invasiveness and chemotherapy resistance. Therefore, this study aimed to evaluate the effects of ALDH inhibition in endometrial CSCs. ECC-1 and RL95-2 cells were submitted to a sphere-forming protocol to obtain endometrial CSCs. ALDH inhibition was evaluated through ALDH activity and expression, sphere-forming capacity, self-renewal, projection area, and CD133, CD44, CD24, and P53 expression. A mass spectrometry-based proteomic study was performed to determine the proteomic profile of endometrial cancer cells upon N,N-diethylaminobenzaldehyde (DEAB). DEAB reduced ALDH activity and expression, along with a significant decrease in sphere-forming capacity and projection area, with increased CD133 expression. Additionally, DEAB modulated P53 expression. Endometrial cancer cells display a distinct proteomic profile upon DEAB, sharing 75 up-regulated and 30 down-regulated proteins. In conclusion, DEAB inhibits ALDH activity and expression, influencing endometrial CSC phenotype. Furthermore, ALDH18A1, SdhA, and UBAP2L should be explored as novel molecular targets for endometrial cancer.

## 1. Introduction

Endometrial cancer ranks as the second most common gynaecological malignancy worldwide and has the sixth highest incidence in females, predominantly affecting postmenopausal women [1]. Early-stage diagnosis is typical for endometrial carcinomas [2]. However, a subset of patients with recurrent and metastatic disease do not respond effectively to current treatment modalities [3].

Cancer stem cells (CSCs) are a subpopulation of tumour cells that have an unusual ability for self-renewal and differentiation within the heterogeneity of malignant tumours [4]. CSCs exhibit an undifferentiated phenotype and are believed to play a pivotal role in triggering tumorigenesis, disease progression, recurrence, and metastasis [3,4,5]. CSC populations have been identified in several types of tumours, including endometrial cancer, based on the expression of stemness-associated markers [6]. Notably, CD133 and aldehyde dehydrogenase (ALDH) have been highlighted as potential CSC markers [3,7,8,9,10,11,12].

ALDH is a member of the NAD(P)^+^-dependent enzyme family and is involved in the oxidation process of aldehydes to the corresponding carboxylic acids [13]. Additionally, this enzyme participates in retinoic acid metabolism and gamma-aminobutyric acid synthesis, both of which contribute to CSC maintenance and differentiation [14,15]. Remarkably, endometrial adenocarcinomas, characterised by high ALDH activity, display increased invasiveness and tumorigenicity [11] and resistance to chemotherapy [16]. Recent research has identified ALDH1 as an attractive therapeutic target [17] and as a potential predictor of endometrial cancer progression [18]. Immunohistochemical analysis has shown minimal ALDH1 expression in benign proliferative and secretory endometrium, while cytoplasmic ALDH1 expression exhibits a stepwise increase in endometrial hyperplasia, atypical hyperplasia, and endometrial cancer. ALDH1 has also demonstrated early predictive value in endometrial cancer development, suggesting its potential as an independent prognostic indicator [18]. In our previous work, we observed clear enrichment of ALDH1/2, along with self-renewal and differentiation capacity, tumorigenic potential, expression of other stemness-associated markers, preference for oxidative metabolism, and resistance to chemotherapy in CSCs derived from the endometrial cancer cell line ECC-1 [19,20].

While the inhibition of ALDH has been studied in breast, cervical, and ovarian cancers using all-trans retinoic acid (ATRA), N,N-diethylaminobenzaldehyde (DEAB), and JQ1 [17,21,22,23], to the best of our knowledge, the topic of ALDH inhibition in endometrial CSCs remains understudied [24].

Due to the prominent role of ALDH in the endometrial stemness pathways, and its association with chemoresistance mechanisms, as well as the promising results of ALDH inhibition strategies in breast and gynaecological cancers, it is imperative to further investigate the functional significance of this enzyme in endometrial cancer. Therefore, the primary objective of this study was to assess the effects of ALDH inhibition in endometrial CSC. The findings from this study may contribute to rationalising novel molecular therapeutic approaches targeting endometrial cancer stemness pathways.

## 2. Materials and Methods

### 2.1. Cell Cultures

The human endometrioid endometrial cancer cell lines ECC-1 (CRL-2923) and RL95-2 (CRL-1671) were acquired from the American Type Culture Collection (ATCC, USA). The cells were cultured and propagated according to the provider’s recommendations at 37 °C and in a 5% CO_2_ atmosphere. For RL95-2 cell culture, a 1:1 mix of Dulbecco’s Modified Eagle Medium (DMEM, Sigma, USA, D5648) and a Nutrient Mixture F-12 Ham (F12, Sigma, USA, N6760), supplemented with 10% foetal bovine serum (FBS, Sigma, USA, F-7524, non-USA origin), 0.005 mg/mL human insulin (Sigma, USA, I0516), 100 mM sodium pyruvate, and 1% antibiotic, was used. ECC-1 cancer cells were propagated as described [19]. Adherent cultures were detached with 0.25% trypsin–EDTA (Sigma, USA, T4049) to prepare cell suspensions for further studies.

### 2.2. Sphere-Forming Protocol

A 5-day sphere-forming protocol was performed to obtain endometrial CSC-enriched populations, as detailed in a previous publication [20]. Spherical colonies of cancer cells, referred to as RL95-2 CSCs and ECC-1 CSCs, were collected.

### 2.3. ALDH Inhibition

ATRA (SC-200898, Santa Cruz Biotechnology, Inc., USA), DEAB (SC-238831, Santa Cruz Biotechnology, Inc., USA), and JQ1 (Sigma, USA, SML1524) were used as ALDH inhibitors. To ensure the absence of cytotoxicity, cell cultures were incubated for 48 h with a range of concentrations, as determined by previously published studies [21,22,23].

CSC-enriched populations were obtained under ALDH inhibition. For this, the sphere-forming protocol was performed in the presence of 100 µM DEAB (added to the culture medium used in the sphere-forming protocol at the time of plating).

### 2.4. Metabolic Activity

The cytotoxicity of ALDH inhibitors (ATRA, DEAB, and JQ1) in endometrial cancer cells (ECC-1 and RL95-2) was assessed using the MTT (3-(4,5-dimethylthiazol-2-yl)-2,5-diphenyltetrazolium bromide, Sigma, USA, M2128) colourimetric assay. Briefly, cell cultures were incubated with the inhibitors for 48 h. Subsequently, 0.5 mg/mL MTT solution was added to the cells and incubated at 37 °C in a light-free environment for at least 4 h. The formazan crystals were then solubilised (0.04 M hydrochloric acid, Merck Millipore100317, USA, in isopropanol, Sigma, USA, 278475), and absorbance recorded at 570 and 620 nm (Biotek^®^ Synergy HT spectrophotometer, USA).

### 2.5. ALDH Activity

The ALDH activity of ECC-1 CSCs and RL95-2 CSCs was measured through the ALDH Activity Assay Kit (Fluorometric) (ab155894, Abcam, UK) following the manufacturer’s instructions. The sample dilution used was 1:8, and samples were measured in kinetic mode until the endpoint (110 min).

### 2.6. Western Blot

ALDH and P53 expression were evaluated using Western blot. Total protein extracts were prepared with a radioimmunoprecipitation assay buffer (RIPA), supplemented with a cocktail of protease inhibitors (cOmplete Mini, Roche, Germany, 118 361 700 01) and 1 mM of dithiothreitol (DTT, Sigma, USA, 43815). For the protein quantification, the bicinchoninic acid method was used (BCA protein assay kit, Pierce™, USA).

For these experiments, 10% acrylamide gels (0.1% SDS) were used. The primary antibodies used for detecting ALDH and P53 were the anti-ALDH 1/2 H-8 (detecting ALDH1A1, ALDH1A2, ALDH1A3, and ALDH2 isoforms of mouse, rat, and human origin) prepared in mice (Santa Cruz Biotechnology, Inc., SC166362, USA), anti-P53 DO7 (Santa Cruz Biotechnology, Inc., SC47698, USA), and anti-Actin (Sigma-Aldrich, Israel). An appropriate anti-mouse secondary antibody (GE Healthcare, UK, RPN5781) was used. Polyvinylidene difluoride membranes (Trans-Blot Turbo RTA Mini 0.2 µm PVDF Transfer Kit, for 40 blots #1704272, BioRad, USA) were incubated with an enzyme substrate (ECF Western blotting Reagent Pack, Amersham Biosciences, United Kingdom) and imaged in a fluorescence scanner (Typhoon 9000 FLA, GE Healthcare Bio-Sciences AB, Sweden). The results obtained were analysed using Image J software v.1.50.

### 2.7. Sphere-Forming Capacity, Self-Renewal, Projection Area, and Differentiation

Endometrial cancer cells were characterised by their ability to form spheres (cell lines’ ability to form CSC colonies) and self-renewal capacity (CSC-derived single cells’ ability to form new CSC colonies with a diameter larger than 40 µm), as previously described [20]. For sphere-forming capacity experiments, 1 × 10^6^ cells were seeded in T25 suspension flasks. Regarding self-renewal, obtained spheres were dissociated in single cells, and 8 × 10^5^ cells were distributed per each 3 mL of sphere medium culture in T25 suspension flasks. The percentage of obtained spheres for both evaluations was calculated through the ratio of the number of spheres obtained vs. the number of seeded cells.

At least six random images per condition were acquired from the plates where spheres were obtained, using a Nikon Eclipse TS 100 microscope (Nikon, Japan) with a Moticam 1080 camera equipped with Motic Images Plus 3.0 software (Motic, Germany). The images were processed using Image J software by delineating the areas of the spheres and obtaining the total area in pixels to obtain the projection area [20].

After CSC-enriched colony dissociation, the sphere-derived cells were cultured in adherent conditions using the culture media of the parental cancer cell lines to assess the obtained differentiated cells (denoted as RL95-2 G1).

### 2.8. Flow Cytometry

Cell suspensions were labelled and analysed using flow cytometry to assess the expression of stemness-associated markers CD24, CD44, and CD133 following the detailed protocol described [20]. The antibodies used for labelling were allophycocyanin-H7-conjugated anti-CD24 (APC-H7 Clone ML5; BD Biosciences, USA), pacific blue conjugated anti-CD44 (PB; Biolegend, 103020), and allophycocyanin conjugated CD133 (APC; Miltenyi Biotec, USA, 293C3-APC), as per the instructions provided by the suppliers. The analysis was performed using the FACS-Canto II cytometer (BD, USA) with the FACSDiva software, version 6.1.2 (BD, USA). The results were presented as the mean fluorescence intensity (MFI).

### 2.9. Peptide Digestion

A protein solution containing sodium dodecyl sulphate (SDS) and dithiothreitol (DTT) was loaded into filtering columns and washed exhaustively with 8 M urea in HEPES buffer. Proteins were then incubated overnight with trypsin sequencing grade (Promega, Madison, USA) after reduction with DTT and alkylation with iodoacetamide (IAA) [25]. The digested peptides (supernatant) were collected by centrifuging for 20 min at 14,000 rpm at 4 °C.

### 2.10. Mass Spectrometry

As previously described [26], samples were analysed by mass spectrometry-based proteomics using nano-LC-MSMS equipment (Dionex RSLCnano 3000, Thermo Scientific, UK) coupled to an Exploris 480 Orbitrap mass spectrometer (Thermo Scientific, UK). Briefly, peptide samples were analysed by nano-LC-MS/MS (Dionex RSLCnano 3000) coupled to a Q-Exactive Orbitrap mass spectrometer (Thermo Scientific, UK). The samples (5 µL) were loaded onto a custom-made fused capillary precolumn (length, 2 cm; outer diameter, 360 µm; and inner diameter, 75 µm) with a flow of 5 µL/min for 7 min. Trapped peptides were separated on a custom-made fused capillary column (length, 20 cm; outer diameter, 360 µm, and inner diameter, 75 µm) packed with ReproSil-PurC18 3 µm resin (Dr Maisch, Germany) with a flow of 300 nL/min using a linear gradient from 92% A (0.1% formic acid) to 28% B (0.1% formic acid in 100% acetonitrile) over 93 min followed by a linear gradient from 28 to 35% B over 20 min at a flow rate of 300 nL/min. Mass spectra were acquired in positive ion mode, applying an automatic data-dependent switch among one Orbitrap survey. MS scanning in the mass range of 400 to 1200 mass/charge ratio (*m*/*z*) was followed by higher-energy collisional dissociation (HCD) fragmentation and Orbitrap detection of the 15 most intense ions observed in the MS scan. The target value in the Orbitrap for the MS scan was 1,000,000 ions at a resolution of 70,000 at *m*/*z* 200. Fragmentation in the HCD cell was performed at a normalised collision energy of 31 eV. The ion selection threshold was set to 25,000 counts, and the maximum injection time was 100 ms for MS scans and 300 and 500 ms for MS/MS scans.

### 2.11. Database-Dependent Search of MSMS Data

Mass accuracy was set at 5 ppm on the peptide level and 0.01 Da on the fragment ions. A maximum of four missed cleavages were used. Carbamidomethyl (C) was set as a fixed modification. Methionine oxidation, N-terminal protein acetylation, lysine diglycine tag, lysine acetylation, serine, threonine, and tyrosine phosphorylation were set as variable modifications. The MSMS spectra were searched against a standard human proteome database from UniProt (3AUP000005640) with a concatenation of all the sequences in reverse, maintaining only lysine and arginine in place. The data were searched and quantified with VEMS [27].

### 2.12. Statistical Analysis

GraphPad version 8.0.1 was used. Descriptive analysis included calculating central tendency and dispersion values. Quantitative variables were presented as mean ± standard error.

For the analysis of metabolic activity, comparisons were performed using a one sample *t*-test for the mean, considering a control value of 100%. For the expression of ALDH and P53, comparisons were performed using the one-sample *t*-test for the mean with a normalisation value of 1. For the analysis of sphere-forming capacity, self-renewal, sphere projection area, expression of CD24, CD44, and CD133, and ALDH activity, the one-way ANOVA test was used after confirming normal distribution and homogeneity of variances. If these assumptions were not met, a Kruskal–Wallis test was performed. Recommended correction methods were used to correct for multiple comparisons. A significance value of 5% was considered for all comparisons.

For the quantitative proteome analysis, protein ion counts were pre-processed by three approaches: (i) removing common MS contaminants followed by log_2_(x + 1) transformation; (ii) removing common MS contaminants followed by log_2_(x + 1) transformation and quantile normalisation; and (iii) removing common MS contaminants followed by log_2_(x + 1) transformation, quantile normalisation, and abundance filtering to optimise the overall Gaussian distribution of the quantitative values.

Statistical analysis was performed using the R package “limma” version 4.04 (The R Foundation, Austria) [28], where contrasts between different cell culture conditions were calculated. Correction for multiple testing was applied using the Benjamini and Hochberg method [29]. Venn diagrams were made with the R package “ggVennDiagram” [30]. Enrichment analysis of proteins was calculated using the R function dhyper as previously described [31]. Heatmaps were generated by using the R package “heatmap.3”. Significantly regulated protein isoforms were collapsed to the genes that encode them to facilitate visualisation and functional analysis of regulated proteins [32].

## 3. Results

### 3.1. RL95-2 CSC-Enriched Populations Formed Spherical Colonies with Self-Renewal Capacity, Differentiation Ability, and Expression of Stemness-Associated Markers

Endometrial cancer cells RL95-2 subjected to the sphere-forming protocol exhibited the formation of high-density spherical suspended colonies characterised by irregular borders. As depicted in Figure S1a, these colonies, identified as RL95-2 CSCs, demonstrated the capability to differentiate under adherent conditions. The resulting monolayer displayed a morphology closely resembling that of the parental RL95-2 cell line. For ease of comprehension, these differentiated cells are hereafter referred to as RL95-2 G1. The RL95-2 cell line presented a sphere-forming capacity of 1.27 ± 0.08% (Figure S1b), and single cells obtained from the spheres showed a self-renewal of 0.36 ± 0.07% (Figure S1c). RL95-2 CSCs presented a projection area of 4.40 × 10^5^ ± 1.95 × 10^4^ pixels (Figure S1d). Stemness-associated markers CD44, CD24, CD133, and ALDH were examined (Figure S1e–h). Four distinct cell populations were identified, labelled CD44^+^/CD24^−^, CD44^weak^/CD24, CD44^weak^/CD24^±^, and CD44^−^/CD24^+^ (Figure S1f). The typically high CD44 expression associated with low CD24 expression [33] was observed in significantly higher amounts in RL95-2 CSCs (37.13 ± 3.83%) than in the parental cell line (12.22 ± 4.81%, *p* = 0.027) and RL95-2 G1 (10.00 ± 2.28%; *p* < 0.001), as quantified in Figure S1f. CD133 expression varied among these populations (Figure S1g). While high CD133 was observed in the CD44^weak^/CD24^−^ RL95-2 and G1 cells, RL95-2 CSCs presented high CD133 not only in the CD44^weak^/CD24^−^ population, but also in the CD44^+^/CD24^−^. Other populations exhibited low or no CD133. ALDH expression (Figure S1h) tended to be higher in RL95-2 CSCs (1.19 ± 0.08) compared to RL95-2 cells and G1 (0.69 ± 0.13). P53 expression significantly decreased in CSCs compared to RL95-2 cells (Figure S1i), with a value of 0.47 ± 0.03 (*p* = 0.047). RL95-2 G1 also exhibited a decrease in P53 expression compared to the parental cell line, with a value of 0.65 ± 0.15.

### 3.2. Safe Concentrations of ATRA, DEAB, and JQ1 Preserved Metabolic Activity, with 100 µM DEAB Inhibiting ALDH Expression in ECC-1 Cells

The impact of varying concentrations of ATRA, DEAB, and JQ1 on the metabolic activity of ECC-1 and RL95-2 cell lines is depicted in Figure S2. The majority of tested concentrations proved non-cytotoxic to endometrial tumour cells, preserving metabolic activity. For example, ECC-1 cells exhibited reduced metabolic activity at a high ATRA concentration of 50 µM (39.47 ± 4.84%; *p* < 0.001). The most elevated DEAB concentrations led to a significant decrease in metabolic activity in RL95-2 (250 µM, *p* = 0.002; 500 µM, *p* < 0.001) and ECC-1 (500 µM, *p* < 0.001). Overall, concentrations such as up to 20 µM for ATRA, 250 µM for DEAB, and 500 nM for JQ1 were deemed safe without compromising the metabolic activity of both cell lines.

Complementarily, the influence of the ALDH inhibitors on their expression was assessed (Figure S3). ECC-1 cells displayed reduced ALDH expression following treatment with 10 µM ATRA, 50 to 250 µM DEAB, and 500 nM JQ1. RL95-2 cells exhibited decreased ALDH expression only with 10 µM ATRA treatment. Considering the absence of observed cytotoxicity with DEAB up to 100 µM in both cell lines and the apparent inhibition of ALDH expression, this concentration was selected for subsequent studies.

### 3.3. DEAB Decreased the ALDH Activity and Expression of Endometrial CSC

**Figure 1:**
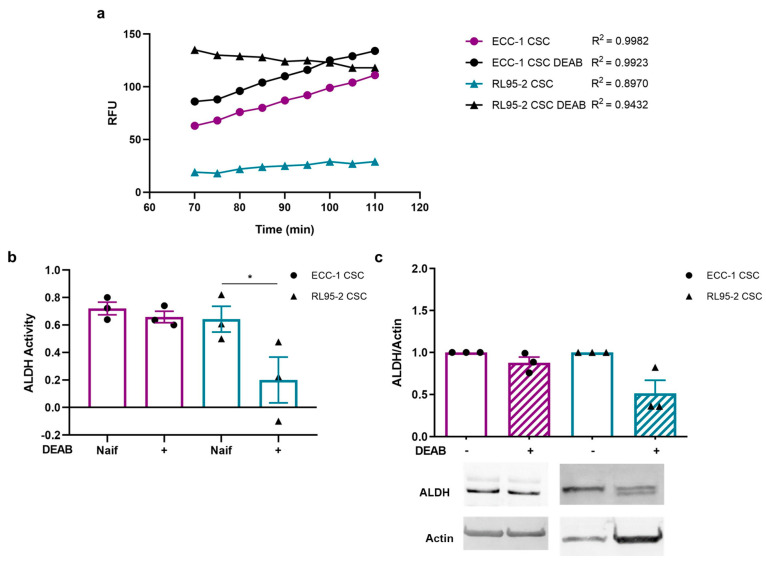
Aldehyde dehydrogenase (ALDH) activity and expression on ECC-1 and RL95-2 cancer stem cells (CSCs) obtained under and in the absence of N,N-diethylaminobenzaldehyde (DEAB). (**a**) Relative fluorescence units (RFUs) obtained over 40 min of the ALDH activity analysis kit reaction (Abcam’s PicoProbe ALDH Activity Assay Kit). (**b**) Variation in the ALDH activity over 40 min of reaction. The results represent the mean and standard error of three samples in two independent experiments. Statistical significance is represented with * for *p* < 0.050. (**c**) Expression of ALDH1/2. The results correspond to the ratio between the fluorescence intensities of ALDH1/2 and β-actin (ratio equal to 1). The results presented correspond to the mean and standard error of three independent experiments. Original western blots are contained in Supplementary File S1.

CSC ALDH activity is presented in Figure 1a,b. Figure 1a illustrates the kinetic analysis of ALDH activity over 40 min, measured in Relative Fluorescence Units (RFU), using an ALDH activity analysis kit. The graph illustrates linear trajectories with varying slopes, indicative of the dynamic nature of ALDH enzymatic activity. ECC-1 CSCs exhibited a steady increase, while ECC-1 CSCs obtained under DEAB, parallel to the untreated counterpart, displayed a slightly elevated trend. Intriguingly, RL95-2 CSCs displayed a unique trajectory, starting from a lower baseline and concluding at a reduced fluorescence intensity. Still, RL95-2 obtained under DEAB displayed a reduction in ALDH activity, exhibiting a gradual decline through the reaction period (Figure 1a). These results translate into Figure 1b. RL95-2 CSCs, influenced by DEAB, exhibited a significant decrease in ALDH activity, registering a value of 0.20 ± 0.17, in contrast to RL95-2 CSCs without DEAB at 0.64 ± 0.09 (*p* = 0.043) (Figure 1b). Interestingly, ECC-1 CSCs under DEAB influence (0.66 ± 0.04) demonstrated comparable activity to ECC-1 CSCs without the inhibitor (0.72 ± 0.05), along with a similar ALDH1/2 expression (0.88 ± 0.07). However, RL95-2 CSCs under DEAB influence also exhibited reduced ALDH1/2 expression (0.52 ± 0.15) (Figure 1c).

### 3.4. ALDH Inhibition Decreases CSC Colony Size, Number, and Self-Renewal, with a Slight Impact on Stemness-Associated Markers

**Figure 2:**
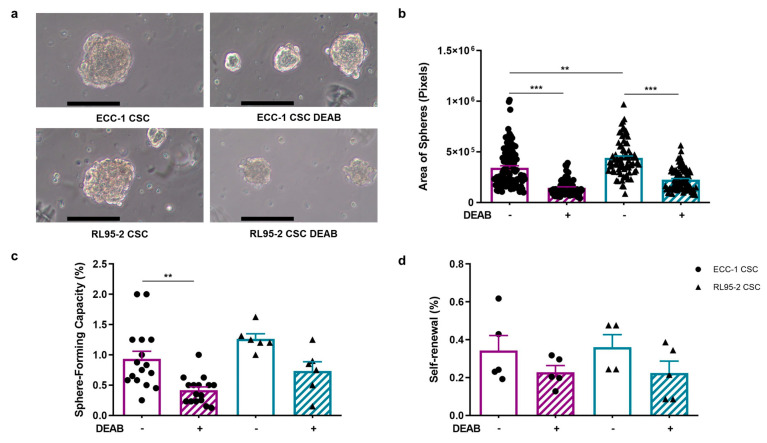
ECC-1 and RL95-2 CSC-enriched population morphology, projection area-forming capacity, and self-renewal and spheres obtained under and in the absence of ALDH inhibition. (**a**) Representative images of the CSC colonies. Images were obtained at 100× magnification. The scale bar represents 100 µm. (**b**) The projection area is expressed as the average number of pixels and the standard error of at least 77 images for each condition, corresponding to at least three independent experiments. (**c**) The sphere-forming capacity is presented as the mean and standard error of at least six independent experiments. (**d**) The self-renewal is represented as the mean and standard error of at least two independent experiments in duplicate. Statistical significance is represented with ** for *p* < 0.010 and *** for *p* < 0.001.

The sphere-forming capacity, self-renewal, and projection area of ECC-1 and RL95-2 spheres are presented in Figure 2. Endometrial CSCs showcased distinct morphological features, with ECC-1 CSCs adopting a spherical and compact structure characterised by regular borders, whereas RL95-2 CSCs displayed a different appearance, featuring a solid structure with irregular borders. CSCs obtained under the influence of DEAB retained their overall structural integrity and morphology; however, they exhibited an evident reduction in size (Figure 2a). The sphere projection area was greater in RL95-2 than in ECC-1 CSCs (*p* = 0.001), measuring 4.40 × 10^5^ ± 1.98 × 10^4^ pixels and 3.43 × 10^5^ ± 2.03 × 10^4^ pixels, respectively (Figure 2b). As anticipated by the morphological evaluation, the mean projection area of CSCs obtained under DEAB influence was significantly smaller for both RL95-2- and ECC-1-origin CSCs, measuring 2.25 × 10^5^ ± 1.13 × 10^4^ pixels (*p* < 0.001) and 1.46 × 10^5^ ± 9.03 × 10^3^ pixels (*p* < 0.001) for RL95-2- and ECC-1-origin CSCs, respectively.

The ECC-1 cell line presented a sphere-forming capacity of 0.93 ± 0.13%, which significantly decreased to 0.42 ± 0.06% (*p* = 0.004) when submitted to 100 µM DEAB (Figure 2c). Regarding the RL95-2 cell line, the ability to form spheres was 1.27 ± 0.08%, and under the influence of the inhibitor, the capacity decreased to 0.73 ± 0.15%. The self-renewal capacity of ECC-1 CSCs was 0.34 ± 0.08%, similar to RL95-2 CSCs (0.36 ± 0.07%). When obtained under the presence of DEAB, the self-renewal presented a tendency to decrease in both CSC populations, being 0.23 ± 0.03% and 0.22 ± 0.06%, for ECC-1 and RL95-2 spheres, respectively (Figure 2d).

**Figure 3:**
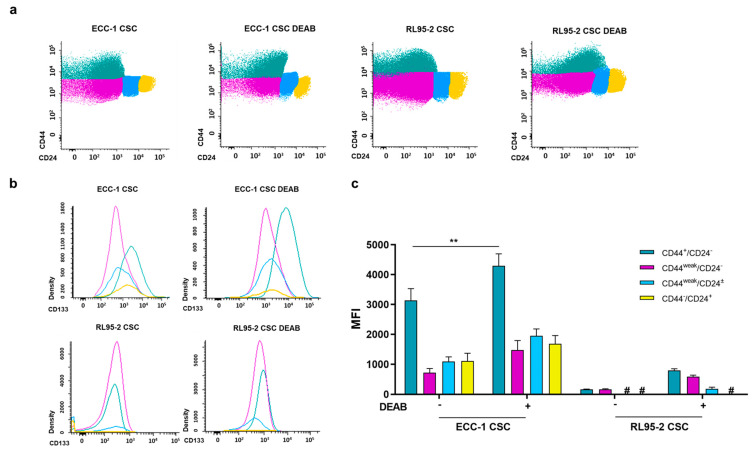
Expression of CD44, CD24, and CD133 of endometrial CSCs obtained under or in the absence of DEAB. (**a**) Representative plots of CD44/CD24. The colours green, pink, blue, and yellow lines correspond to the CD44^+^/CD24^−^, CD44^weak^/CD24^−^, CD44^weak^/CD24^±^, and CD44^−^/CD24^+^ populations, respectively. (**b**) Representative histograms of CD133 expression. The line colours green, pink, blue, and yellow correspond to the CD44^+^/CD24^−^, CD44^weak^/CD24^−^, CD44^weak^/CD24^±^, and CD44^−^/CD24^+^ populations, respectively. (**c**) CD133 expression represented as the mean fluorescence intensity (MFI) and standard error of at least two independent experiments. Statistical significance is represented with ** for *p*<0.010. # represents the absence of expression.

The stemness-associated markers CD44, CD24, and CD133 were examined in the endometrial CSC populations obtained with and without the ALDH inhibitor DEAB (Figure 3). Firstly, four different populations were identified considering CD44 and CD24, which are CD44^+^/CD24^−^, CD44^weak^/CD24^−^, CD44^weak^/CD24^±^, and CD44^−^/CD24^+^. Table 1 outlines the percentage distribution of the abovementioned populations, with CD44^+^/CD24^−^ and CD44^weak^/CD24^−^ being the most prevalent in both ECC-1 and RL95-2 CSCs (see also Figure 3a). Comparing ECC-1 and RL95-2 CSCs, no significant differences were observed in the CD44/CD24 populations, except for the CD44-/CD24^+^, which were higher on ECC-1 (*p* = 0.016). Notably, the presence of 100 µM DEAB during the sphere-forming protocol did not impact the CD44/CD24 phenotype of endometrial CSCs (*p* = n.s.).

CD133 expression varied between these populations, as can be observed in the representative histograms (Figure 3b) and mean fluorescence intensity (MFI) values (Figure 3c). The CD44^+^/CD24^−^ population of ECC-1 CSCs is positive for CD133. With the influence of DEAB, a subtle shift occurs, and the CD44^+^/CD24^−^ subgroup exhibits increased CD133 expression. A trend toward higher CD133 expression was also observed in the RL95-2 CSC CD44^+^/CD24^−^ and CD44^weak^/CD24^−^ populations obtained under the influence of DEAB (Figure 3b).

The expression of CD133, as depicted in Figure 3c as MFI, reveals that RL95-2 CSCs showed almost negligible expression of CD133, irrespective of the presence or absence of DEAB influence. In the CD44^+^/CD24^−^ population, ECC-1 CSCs displayed an MFI of 3140.17 ± 392.08. However, under DEAB treatment, ECC-1 CSCs showed a significant increase in MFI, reaching 4298.67 ± 400.49 (*p* = 0.005) compared to untreated ECC-1 CSCs. For ECC-1 CSCs in the CD44^weak^/CD24^−^ population, CD44^weak^/CD24^±^ population, and CD44^−^/CD24^+^ population, the MFI values were 728.00 ± 138.49, 1094.17 ± 154.12, and 1106.83 ± 263.18, respectively. Interestingly, there was no statistical significance observed in DEAB-treated ECC-1 CSCs, with corresponding values of 1476.50 ± 317.13, 1952.67 ± 228.86, and 1679.67 ± 281.30 MFI.

**Figure 4:**
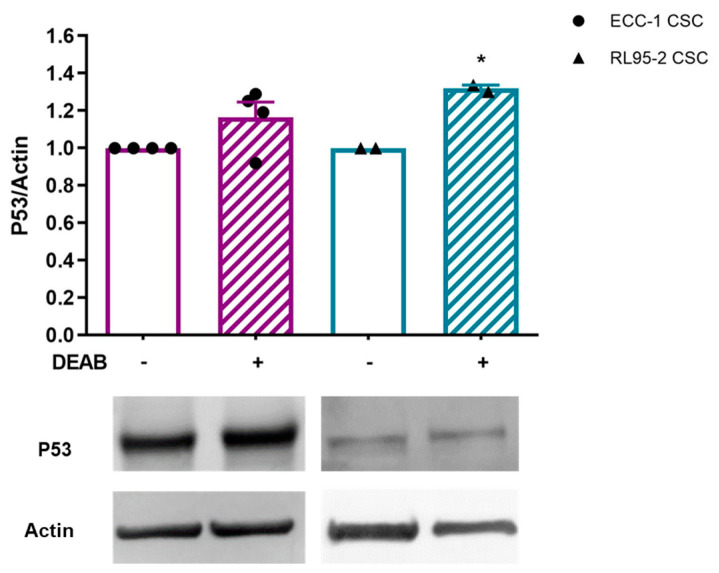
Expression of P53 in endometrial CSCs obtained under or in the absence of DEAB. The results correspond to the ratio between the fluorescence intensities of P53 and β-actin (P53/control actin ratio equal to 1). The results presented correspond to the mean and standard error of at least two independent experiments. Statistical significance is represented with * for *p* < 0.050. Original western blots are contained in Supplementary File S1.

P53 is essential for controlling the self-renewal and differentiation of CSCs. However, ECC-1 CSCs obtained in the presence of DEAB showed a protein expression of 1.16 ± 0.08. In the case of RL95-2 CSCs, a significant increase in P53 expression was observed (1.32 ± 0.02; *p* = 0.036, Figure 4).

### 3.5. ECC-1 and RL95-2 Shared ALDH Isoforms in Their Proteomes

The proteomic profiling of endometrial cancer cell lines revealed that ECC-1 and RL95-2 shared the ALDH isoforms ALDH3A1, ALDH3A2, ALDH3B1, ALDH3B2, ALDH7A1, ALDH9A1, and ALDH18A1 in their proteome. Additionally, ALDH1L1, ALDH6A1, and ALDH16A1 were also present in the proteomic profile of the ECC-1 cell line. In both cell lines, the most abundant protein corresponded to ALDH18A1 (Table S1).

### 3.6. ALDH Inhibition Induced Changes in Protein Regulation of Endometrial Cancer Cells

**Figure 5:**
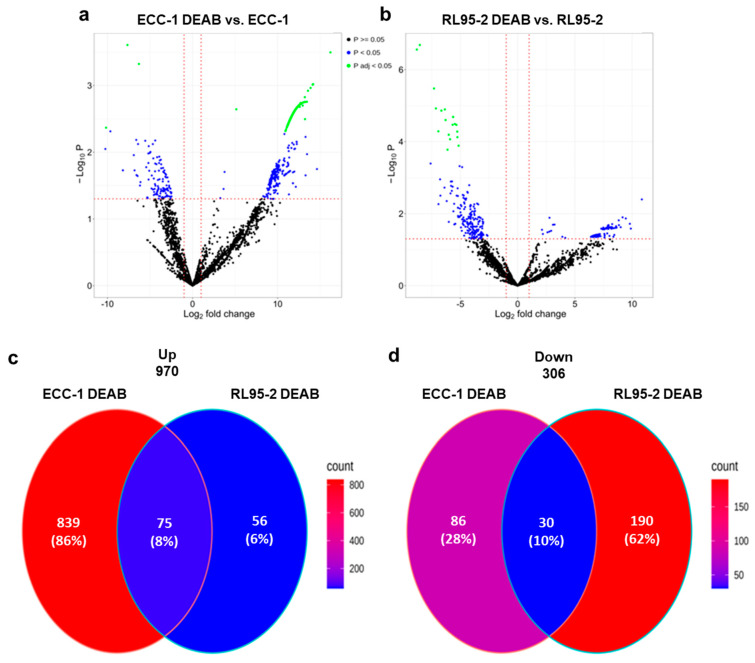
Proteomic profile of endometrial cancer stem cells upon ALDH inhibition. Volcano plot for the comparisons: (**a**) ECC-1 DEAB versus ECC-1 and (**b**) RL95-2 DEAB versus RL95-2. Venn diagrams comparing (**c**) up-regulated proteins in the two cell lines and (**d**) down-regulated proteins in the two cell lines upon DEAB treatment.

The cell lines ECC-1 and RL95-2 were subjected to ALDH inhibition, and their proteomes were profiled by LC-MS. In ECC-1, 327/1030 proteins were regulated with a fold change higher than 1.5 at a 0.05 adjusted *p*-value or 0.05 *p*-value (Table S2 (Proteome of ECC-1 DEAB vs. ECC-1) and Table S3 (Proteome of RL95-2 DEAB vs. RL95-2)). Figure 5a,b present the volcano plots for the two comparisons. A large number of proteins were regulated in both cell lines. However, the proteome responses to ALDH inhibition in the two cell lines appear different in that ECC-1 mainly displays up-regulated proteins, whereas treatment in RL95-2 mainly results in down-regulated proteins. Figure 5c,d depicts Venn diagrams comparing the up- and down-regulated proteins from the two cell lines. Although it is evident that the two cell lines appear to have a unique response given a large number of regulated proteins that are not common in the two cell lines, there is a common response of 105 proteins that display the same direction of regulation. Interestingly, ALDH16A1 was up-regulated in both cell lines upon ALDH inhibition.

Of the down-regulated proteins identified in both cell lines upon DEAB treatment, we identified three proteins as potential biomarkers of endometrial cancer. These proteins are aldehyde dehydrogenase 18A1 (ALDH18A1), succinate dehydrogenase [ubiquinone] flavoprotein subunit (SdhA), and ubiquitin-associated protein 2-like (UBAP2L), corresponding to the most down-regulated proteins shared by both cell lines (Table S4).

**Figure 6:**
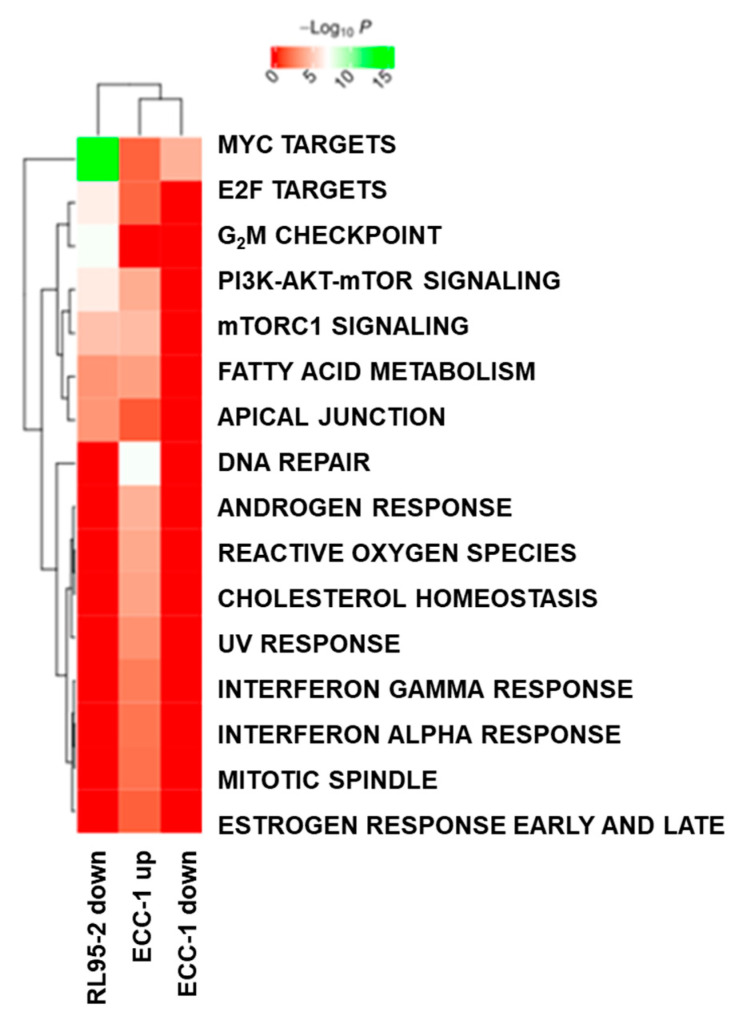
Functional enrichment analysis of regulated proteins in the subgroups defined by the Venn diagrams in Figure 5.

To obtain an overview of the functional regulation for the subgroups defined in the Venn diagrams in Figure 5c,d, functional enrichment against cancer hallmark proteins was performed (Figure 6). When matched against cancer hallmark proteins, the protein intersections shown in Figure 5, as well as protein only up-regulated in RL95-2, did not result in significant enrichment. However, both ECC-1 and RL95-2 down-regulated proteins were enriched in “Myc targets”. In addition, RL95-2 down-regulated proteins displayed a highly significant enrichment in G_2_M checkpoint proteins. ECC-1 up-regulated proteins showed the highest functional enrichment for DNA repair proteins.

## 4. Discussion

The ALDH1A1 isoform plays a pivotal role in maintaining CSC phenotype, underscoring its potential as a therapeutic target in cancer [13]. To understand the influence of ALDH on endometrial stemness, this study investigated ALDH inhibition on endometrial CSCs.

ALDH inhibition using ATRA, DEAB, and JQ1 was previously achieved in breast and ovarian cancer [21,22,23]. In breast cancer cells, 5 μM ATRA or 100 μM DEAB maintained cell proliferation and viability [21]. The same was observed in ovarian cancer cells after exposure to 5 µM ATRA and 50 µM DEAB for 48 h [22]. Still, JQ1 exhibited IC50 values up to 1 μM, according to the cell line [34]. In the case of endometrial cancer cells, viability persisted after exposure to ALDH inhibitors, except under high concentrations (Figure S2). Regarding ALDH expression, incubation with inhibitors revealed nuanced responses. ATRA diminished ALDH expression in both cell lines, but we saw a concurrent elevation of metabolic activity. Moreover, ATRA is known to interfere with multiple pathways beyond our study’s scope [35,36,37]. In OVCAR3, an ovarian cancer line, JQ1 treatment at 50–100 nmol/L resulted in a dose-dependent decrease in ALDH expression [23]. JQ1 did not outperform DEAB in terms of ALDH expression levels in RL95-2 cells. Nevertheless, while DEAB exhibited a seemingly negligible impact on enzyme expression in RL95-2 cells, ECC-1 cells demonstrated a reduction in ALDH expression upon DEAB treatment (Figure S3). Although JQ1 showed a potential ALDH inhibition effect, which should be further explored, its efficacy was seen alongside an unexpected increase in ALDH expression at low inhibitor concentrations. Considering these insights and the initial results, 100 μM DEAB was selected for the subsequent studies.

In this study, endometrial CSC-enriched cultures were obtained through a sphere-forming protocol optimized for gynaecological tumours. The protocol involved a cell culture medium containing methylcellulose and lacking foetal bovine serum (FBS) to maintain non-adherent conditions. Additionally, the medium was supplemented with growth factors to support the undifferentiated phenotype of cells [20]. Spheres obtained from the RL95-2 cell line originated adherent-derived cells in a monolayer (Figure S1a), like we previously showed for the ECC-1 cell line [19], confirming the differentiation ability of CSC populations [38].

The ALDH overexpression we previously observed in endometrial CSCs (ECC-1 CSCs) pointed out this enzyme as a promising therapeutic target for endometrial cancer [19]. In the RL95-2 CSC population, there is a noticeable trend toward increased ALDH expression (Figure S1h). This finding aligns with the recognised role of ALDH as a CSC marker, supported by similar results in other studies. For instance, ALDH1 expression was significantly higher in CD133^+^ cells isolated from an endometrial tumour sample than in the CD133^−^ cell population [39]. In fact, high ALDH activity has been widely recognised as a marker of cancer stem cells and is commonly employed in flow cytometry studies for their identification and isolation [40]. ALDH activity was similar in RL95-2 CSCs and ECC-1 CSCs (Figure 1b). A comprehensive study involving multiple lines of endometrioid-type endometrial carcinoma demonstrated increased ALDH activity in HEC-1, HEC-1A, HEC-108, HEC-116, HEC-6, HEC-88nu, and SNG-M. However, ALDH activity was not observed in HEC-251 and SNG-II. These findings underscore variations in the expression and activity of stemness-associated markers within the same type of tumour [11].

Regarding ALDH inhibition, our data suggested decreased ALDH activity in CSC-enriched populations in the presence of DEAB. This was significant in RL95-2 CSCs (Figure 1b). Similarly, a study involving endometrial spheroids enriched with CSCs reported that DEAB inhibited ALDH activity, leading to a decrease in spheroid proliferation [41]. Other research employing DEAB to inhibit ALDH characterises the action of this compound as specific, effectively blocking the enzyme’s activity [21]. In breast CSC populations with high ALDH activity and CD44 expression, DEAB treatment increased sensitivity to chemo- and radiotherapy [21]. Additionally, breast CSCs pre-treated with DEAB exhibited decreased colony-forming ability, suggesting a specific blockade of ALDH activity by DEAB [21]. In addition to the influence on ALDH activity, our findings also indicated a trend towards a decrease in ALDH expression after DEAB treatment (Figure 1c). Moreover, some samples suggested that a potential partial degradation of the enzyme may have occurred.

Considering the results obtained with endometrial cancer cell lines (Figure S3) and CSC populations (Figure 1) upon DEAB treatment, it is evident that there is a contrasting direction regarding ALDH inhibition. The transition from a 2D cell culture to a spheroid culture signifies a shift from a differentiated culture to a CSC-enriched population. Indeed, this occurrence likely mirrors unique cell populations and biological environments inherent to these models. In monolayer cultures, there was a decrease in ALDH expression upon DEAB treatment, and it is worth noting that a reduction in activity may also be occurring concurrently in both cell lines. Interestingly, our findings in 3D cultures unveil unexpected results in comparison to the monolayer cultures. RL95-2 3D cultures exhibit reduced ALDH expression and activity, hinting at a potential transition toward a less stem-like phenotype. Conversely, ECC-1 3D cultures did not demonstrate a significant reduction in ALDH expression or activity. This suggests that despite the initial expectation of decreased ALDH expression and activity in ECC-1 cells in 3D cultures, compensatory mechanisms or alternative stemness pathways might be involved, especially considering the phenotype illustrated in Figure 2. Moreover, the general regulation of proteins and, particularly, ALDH isoforms could vary in endometrial 3D cultures compared to 2D cultures, potentially impacting the efficacy of DEAB inhibition. Effectively, our proteomic findings revealed a different protein signature between both endometrial cancer cell lines.

The proteomic profiles of ECC-1 and RL95-2 showed that ALDH3A1, ALDH3A2, ALDH3B1, ALDH3B2, ALDH7A1, ALDH9A1, and ALDH18A1 are presented in their proteomes, as well as ALDH1L1, ALDH6A1, and ALDH16A1 in ECC-1 cells, ALDH18A1 being the most abundant isoform. Although we used an anti-ALDH validated for isoforms 1A1, 1A2, 1A3, and 2, and these isoforms were not present in the analysis carried out in the proteomic profile of the endometrial cancer cell lines used in this study, it was possible to obtain expression of the enzyme through Western blot. We consider that this may be because the amino acid sequence of the ALDH1A1 isoform, for which the antibody shows affinity, presents a similarity with the total amino acid sequences of the ALDH isoforms present in the proteome of endometrial cancer cells (Table S1).

Both endometrial cancer cell lines presented the ability to form spheres and self-renewal in the presence and absence of DEAB (Figure 2a,c,d). This was consistent with our findings regarding RL95-2 (Figure S1b,c) and ECC-1 sphere-forming and self-renewal ability [19]. In line with this, breast cancer cell lines MCF7 and HCC1806, obtained with the same sphere-forming protocol, showed a sphere-forming capacity of 1.97% and 1.25%, respectively [42]. In an eight-day sphere-forming protocol, the number of spheres formed by ECC-1 and RL95-2 was comparable [43]. Notably, DEAB exposure resulted in a meaningful reduction in the sphere-forming capacity of endometrial cell lines, which attained significance for ECC-1 (Figure 2c). Furthermore, endometrial spheres obtained under DEAB influence during the five-day sphere-formation protocol exhibited a significant decrease in the projection area (Figure 2b), suggesting a potential impact on CSC proliferation. Similar effects of DEAB on the proliferation and colony-forming capacity were observed in pancreatic cells [44].

CD133 has been explored as a potential marker of CSC populations in endometrial cancer [7,19,45]. In the present research, an absence of CD133 expression in the RL95-2 populations was observed (Figure S1g). While certain studies indicate that CD133 expression might be characteristic of CSCs, others show that CD133 is not a universal marker for endometrial CSCs. In the RL95-2 cell line, the CD133^+^ phenotype was found in less than 1% of cells [9]. Similarly, the absence of endometrial cancer cells with CD133^+^/CD44^+^ phenotype has been described [46].

Regarding CD44 and CD24 (Figure S1e,f), the predominant populations exhibited CD44^+^/CD24^−^ and CD44^weak^/CD24^−^ phenotypes, proving the enrichment of CSC phenotype in endometrial spheres. Other studies in endometrial cancer have reported CD44 expression [45]. Ishikawa CSCs, obtained through the sphere-forming colonies, revealed a relevant expression of a CD44^+^/CD133^+^ population [46]. The expression of CD44/CD24 remained unchanged when the endometrial CSCs were obtained in the presence of DEAB (Table 1 and Figure 3a). Notably, our results revealed that when ECC-1 CSCs were obtained under DEAB influence, there was a notable increase in CD133 expression within the CD44^+^/CD24^−^ population, indicating an enhanced CSC phenotype (Figure 3b,c). These data suggest that ALDH and CD133 can be a set of markers for endometrial CSCs, likewise in ovarian CSCs [47]. In further studies, it would be crucial to understand if ALDH inhibition can trigger the enhancement of other CSC markers to counterbalance its inhibition [48].

The RL95-2 cell line presents a mutant P53 expression with a codon deletion in exon 6 [49], a condition that leads to a loss of function of wild-type P53 [50]. Furthermore, in cancer cells, mutations leading to a P53 inactivation could entail an enhancement of stemness-associated markers, as well as the capacity for sphere forming [50], as our results demonstrate. A correlation between ALDH1 and CD44 expression and P53 missense mutations in colorectal cancer suggested a role in disease development and the presence of CSCs [51]. Also, our data revealed that RL95-2 CSCs presented a meaningful reduction in P53 expression when compared to the parental cell line (Figure S1i). Upon ALDH inhibition, endometrial CSCs displayed a trend towards increased P53 expression (Figure 4). These findings suggest that DEAB may modulate P53 expression, potentially influencing a pathway of differentiation.

The discovery of new biomarkers may allow an early and more effective diagnosis, prognostic stratification, or even the development of targeted therapies [52]. In the present study, a mass spectrometry-based proteomic strategy guided by ALDH inhibition in ECC-1 and RL95-2 cell lines was used to find new endometrial cancer markers. As expected, both endometrial cancer cells showed the regulation of a considerable number of proteins after treatment with DEAB. Despite this, ECC-1 and RL95-2 only shared 105 proteins regulated in the same way, revealing tumour heterogeneity and a unique proteomic signature of tumours (Figure 5). In Attarha and collaborators’ study, three endometrial cancer proteome profiles were compared regarding the proteins identified, showing a high level of variability in the origin of proteins. These variations were considered relevant for endometrial tumorigenesis [53]. Additionally, the isoform ALDH18A1 was identified as the most abundant ALDH isoform in the proteomic profile of both endometrial cancer cell lines and one of the most down-regulated proteins upon DEAB treatment, this isoform being considered the most inhibited. Moreover, it was possible to identify three proteins as potential biomarkers for endometrial cancer, namely, ALDH18A1, SdhA, and UBAP2L. According to the Human Protein Atlas, a high expression of ALDH18A1 in endometrial cancer was associated with a better prognosis, reflecting a greater probability of survival [54,55]. In ovarian cancer, overexpression of SdhA is common in ovarian carcinoma patients and was associated with high mitochondrial metabolism in cancer models [56]. The expression of UBAP2L was associated with a poor prognosis in cervical cancer [57], having been associated with the activation of the Wnt/β-catenin signalling pathway in gastric cancer [58] and with the regulation of PI3K/AKT and P53 signalling pathways in hepatocellular carcinoma [59]. Moreover, UBAP2L has been proposed as a potential therapeutic target for gastric cancer [58]. In our understanding, in endometrial cancer the ALDH isoform ALDH18A1 can be a promising prognostic factor. Concerning SdhA and UBAP2L, their involvement in the regulation of stemness-associated markers must be explored in endometrial cancer, which may reveal potential therapeutic targets.

Promising results were obtained regarding the analysis of functional regulation, where both endometrial cancer cell lines demonstrated down-regulated proteins enriched in “Myc targets” upon DEAB treatment (Figure 6). Myc proteins are involved in various cellular domains, such as cell growth, differentiation processes, DNA repair, and stem cell development, among others [60]. These results may indicate a relevant role of DEAB in the control of these cellular processes. A similar result was obtained with RL95-2 down-regulated proteins of the G_2_M checkpoint pathway, given that these proteins are associated with cell proliferation and were correlated with poor survival rates in pancreatic cancer patients [61]. This finding indicated that DEAB seems to interfere with cell proliferation and cell cycle, corroborating the influence exerted in the endometrial CSC profile. Considering the role of DEAB in mutant P53 expression of endometrial CSCs, we hypothesise that this down-regulation in proteins associated with cell cycle and DNA repair induced by DEAB may occur due to alternative P53-independent pathways.

Regarding ECC-1, an up-regulation of DNA repair proteins may suggest that DEAB can promote cell cycle arrest and several repair pathways through the positive regulation of these proteins [62].

The role of DEAB in ALDH inhibition can be controversial. Despite DEAB presenting specificity to several ALDH isoforms, namely, ALDH1A1, 1A2, 1A3, 1B1, 2, 3A1, and 5A1, it presents high selectivity for ALDH1A1 and 3A1, revealing a limited selectivity for other isoforms [17,63]. Regarding these isoforms, DEAB presents its highest sensitivity for ALDH1A1, followed by ALDH2 [64], being considered an irreversible inhibitor [65]. In our study, the ECC-1 cell line presented in its proteome isoforms for which DEAB has specificity (ALDH1B1, 3A1); however, the efficacy of DEAB inhibition may be limited by other isoforms presented in its proteome. Additionally, although DEAB can be considered an ALDH inhibitor [65], it can also act as an ALDH substrate [64]. In the context of our results, these data can be relevant for understanding the ineffectiveness of DEAB in ALDH activity and expression, particularly for ECC-1 CSCs.

In future studies, from a clinical perspective, it would be important to assess the role of DEAB or other ALDH-interfering agents in sensitising endometrial cancer cells and CSCs to chemotherapy and other currently available therapies. Furthermore, the proteomic profile of endometrial CSCs inhibited with DEAB must be determined, to explore new biomarkers for endometrial cancer, namely, for prognosis and the development of innovative therapies targeting CSC pathways.

## 5. Conclusions

DEAB demonstrated a significant impact on ALDH activity, leading to a subsequent reduction in ALDH expression, sphere-forming capacity, self-renewal, and the projection area of endometrial CSCs. Proteomic analysis revealed 105 common proteins altered in ECC-1 and RL95-2 cells upon DEAB treatment, suggesting ALDH18A1, SdhA, and UBAP2L as potential markers for endometrial cancer. Moreover, ALDH18A1 isoform seems to be the most inhibited ALDH isoform in endometrial cancer, and a promising prognostic biomarker.

Although the precise mechanism of DEAB action remains unclear, this study underscores its interference with CSC phenotype. Insights into this pathway could contribute to refining conventional treatments and developing targeted therapies for endometrial CSCs.

## Figures and Tables

**Table 1:** Percentage distribution of CD44/CD24 populations of ECC-1 and RL95-2 cancer stem cells (CSCs) obtained in the presence or absence of N,N-diethylaminobenzaldehyde (DEAB). Presented values express the mean and standard error of at least two independent experiments.

%	ECC-1 CSC	ECC-1 CSC DEAB	RL95-2 CSC	RL95-2 CSC DEAB
CD44^+^/CD24^−^	35.72 ± 5.89	40.93 ± 10.76	37.13 ± 3.83	33.33 ± 4.17
CD44^weak^/CD24^−^	33.45 ± 5.29	29.23 ± 5.43	46.98 ± 3.99	44.40 ± 5.90
CD44^weak^/CD24^±^	23.33 ± 1.90	23.15 ± 5.40	13.15 ± 2.72	17.78 ± 2.09
CD44^−^/CD24^+^	10.32 ± 1.88	6.72 ± 1.29	2.75 ± 1.35	4.50 ± 1.74

## Data Availability

Data obtained within the scope of this study are available in this article or in the Supplementary material. The mass spectrometry proteomics data that support the findings of this study have been deposited in ProteomeXchange Consortium [66] via the PRIDE [67] partner with the PXD052364 accession code.

## Supplementary Materials

The following supporting information can be downloaded at: https://www.mdpi.com/article/10.3390/cancers16112031/s1, Figure S1: Characterisation of endometrial cancer cells. Figure S2: Evaluation of the metabolic activity of human endometrial cancer cell lines, ECC-1 and RL95-2, after 48 h of incubation with ALDH inhibitors. Figure S3: Expression of ALDH in ECC-1 and RL95-2 cells treated with ATRA, DEAB, and JQ1. Table S1: Identified ALDH isoforms in endometrial cancer cell lines. Table S2: Proteome of ECC-1_DEABvsECC-1. Table S3: Proteome of RL95-2_DEABvsRL95-2. Table S4: Identified down-regulated proteins in ECC-1_DEAB and RL95-2_DEAB; File S1: Original western blots.

## References

[1] Sung H., Ferlay J., Siegel R.L., Laversanne M., Soerjomataram I., Jemal A., Bray F. (2021). Global Cancer Statistics 2020: GLOBOCAN Estimates of Incidence and Mortality Worldwide for 36 Cancers in 185 Countries. CA Cancer J. Clin..

[2] Alonso-Alconada L., Muinelo-Romay L., Madissoo K., Diaz-Lopez A., Krakstad C., Trovik J., Wik E., Hapangama D., Coenegrachts L., Cano A. (2014). Molecular Profiling of Circulating Tumor Cells Links Plasticity to the Metastatic Process in Endometrial Cancer. Mol. Cancer.

[3] Carvalho M.J., Laranjo M., Abrantes A.M., Torgal I., Botelho M.F., Oliveira C.F. (2015). Clinical Translation for Endometrial Cancer Stem Cells Hypothesis. Cancer Metastasis Rev..

[4] Allegra A., Alonci A., Penna G., Innao V., Gerace D., Rotondo F., Musolino C. (2014). The Cancer Stem Cell Hypothesis: A Guide to Potential Molecular Targets. Cancer Invest..

[5] Kyo S., Kato K. (2015). Endometrial Cancer Stem Cell as a Potential Therapeutic Target. Semin. Reprod. Med..

[6] Islam F., Gopalan V., Smith R.A., Lam A.K.Y. (2015). Translational Potential of Cancer Stem Cells: A Review of the Detection of Cancer Stem Cells and Their Roles in Cancer Recurrence and Cancer Treatment. Exp. Cell Res..

[7] Rutella S., Bonanno G., Procoli A., Mariotti A., Corallo M., Prisco M.G., Eramo A., Napoletano C., Gallo D., Perillo A. (2009). Cells with Characteristics of Cancer Stem/Progenitor Cells Express the CD133 Antigen in Human Endometrial Tumors. Clin. Cancer Res..

[8] Friel A.M., Zhang L., Curley M.D., Therrien V.A., Sergent P.A., Belden S.E., Borger D.R., Mohapatra G., Zukerberg L.R., Foster R. (2010). Epigenetic Regulation of CD133 and Tumorigenicity of CD133 Positive and Negative Endometrial Cancer Cells. Reprod. Biol. Endocrinol..

[9] Nakamura M., Kyo S., Zhang B., Zhang X., Mizumoto Y., Takakura M., Maida Y., Mori N., Hashimoto M., Ohno S. (2010). Prognostic Impact of CD133 Expression as a Tumor-Initiating Cell Marker in Endometrial Cancer. Hum. Pathol..

[10] Mamat S., Ikeda J.-I., Tian T., Wang Y., Luo W., Aozasa K., Morii E. (2011). Transcriptional Regulation of Aldehyde Dehydrogenase 1A1 Gene by Alternative Spliced Forms of Nuclear Factor Y in Tumorigenic Population of Endometrial Adenocarcinoma. Genes Cancer.

[11] Rahadiani N., Ikeda J.I., Mamat S., Matsuzaki S., Ueda Y., Umehara R., Tian T., Wang Y., Enomoto T., Kimura T. (2011). Expression of Aldehyde Dehydrogenase 1 (ALDH1) in Endometrioid Adenocarcinoma and Its Clinical Implications. Cancer Sci..

[12] Giannone G., Attademo L., Scotto G., Genta S., Ghisoni E., Tuninetti V., Aglietta M., Pignata S., Valabrega G. (2019). Endometrial Cancer Stem Cells: Role, Characterization and Therapeutic Implications. Cancers.

[13] Ciccone V., Morbidelli L., Ziche M., Donnini S. (2020). How to Conjugate the Stemness Marker ALDH1A1 with Tumor Angiogenesis, Progression, and Drug Resistance. Cancer Drug Resist..

[14] Ma I., Allan A.L. (2011). The Role of Human Aldehyde Dehydrogenase in Normal and Cancer Stem Cells. Stem Cell Rev. Rep..

[15] Cojoc M., Mäbert K., Muders M.H., Dubrovska A. (2015). A Role for Cancer Stem Cells in Therapy Resistance: Cellular and Molecular Mechanisms. Semin. Cancer Biol..

[16] Van Der Zee M., Sacchetti A., Cansoy M., Joosten R., Teeuwssen M., Heijmans-Antonissen C., Ewing-Graham P.C., Burger C.W., Blok L.J., Fodde R. (2015). IL6/JAK1/STAT3 Signaling Blockade in Endometrial Cancer Affects the ALDHhi/CD126+ Stem-like Component and Reduces Tumor Burden. Cancer Res..

[17] Muralikrishnan V., Hurley T.D., Nephew K.P. (2020). Targeting Aldehyde Dehydrogenases to Eliminate Cancer Stem Cells in Gynecologic Malignancies. Cancers.

[18] Mah V., Elshimali Y., Chu A., Moatamed N.A., Uzzell J.P., Tsui J., Schettler S., Shakeri H., Wadehra M. (2021). ALDH1 Expression Predicts Progression of Premalignant Lesions to Cancer in Type I Endometrial Carcinomas. Sci. Rep..

[19] Carvalho M.J., Laranjo M., Abrantes A.M., Casalta-Lopes J., Sarmento-Santos D., Costa T., Serambeque B., Almeida N., Gonçalves T., Mamede C. (2019). Endometrial Cancer Spheres Show Cancer Stem Cells Phenotype and Preference for Oxidative Metabolism. Pathol. Oncol. Res..

[20] Laranjo M., Carvalho M.J., Serambeque B., Alves A., Marto C.M., Silva I., Paiva A., Botelho M.F. (2020). Obtaining Cancer Stem Cell Spheres from Gynecological and Breast Cancer Tumors. J. Vis. Exp..

[21] Croker A.K., Allan A.L. (2012). Inhibition of Aldehyde Dehydrogenase (ALDH) Activity Reduces Chemotherapy and Radiation Resistance of Stem-like ALDHhiCD44+ Human Breast Cancer Cells. Breast Cancer Res. Treat..

[22] Januchowski R., Wojtowicz K., Sterzyſska K., Sosiſska P., Andrzejewska M., Zawierucha P., Nowicki M., Zabel M. (2016). Inhibition of ALDH1A1 Activity Decreases Expression of Drug Transporters and Reduces Chemotherapy Resistance in Ovarian Cancer Cell Lines. Int. J. Biochem. Cell Biol..

[23] Yokoyama Y., Zhu H., Lee J.H., Kossenkov A.V., Wu S.Y., Wickramasinghe J.M., Yin X., Palozola K.C., Gardini A., Showe L.C. (2016). BET Inhibitors Suppress ALDH Activity by Targeting ALDH1A1 Super-Enhancer in Ovarian Cancer. Cancer Res..

[24] Shiba S., Ikeda K., Suzuki T., Shintani D., Okamoto K., Horie-Inoue K., Hasegawa K., Inoue S. (2019). Hormonal Regulation of Patient-Derived Endometrial Cancer Stem-like Cells Generated by Three-Dimensional Culture. Endocrinology.

[25] Biedka S., Schmidt B.F., Frey N.M., Boothman S.M., Minden J.S., Lucas A. (2021). Reversible Click Chemistry Tag for Universal Proteome Sample Preparation for Top-Down and Bottom-Up Analysis. J. Proteome Res..

[26] Carvalho A.S., Baeta H., Henriques A.F.A., Ejtehadifar M., Tranfield E.M., Sousa A.L., Farinho A., Silva B.C., Cabeçadas J., Gameiro P. (2021). Proteomic Landscape of Extracellular Vesicles for Diffuse Large B-cell Lymphoma Subtyping. Int. J. Mol. Sci..

[27] Carvalho A.S., Ribeiro H., Voabil P., Penque D., Jensen O.N., Molina H., Matthiesen R. (2014). Global Mass Spectrometry and Transcriptomics Array Based Drug Profiling Provides Novel Insight into Glucosamine Induced Endoplasmic Reticulum Stress. Mol. Cell. Proteom..

[28] Smyth G.K. (2004). Linear Models and Empirical Bayes Methods for Assessing Differential Expression in Microarray Experiments. Stat. Appl. Genet. Mol. Biol..

[29] Benjamini Y., Hochberg Y. (1995). Controlling the False Discovery Rate: A Practical and Powerful Approach to Multiple Testing. J. R. Stat. Soc. Ser. B (Methodol.).

[30] Gao C.H., Yu G., Cai P. (2021). GgVennDiagram: An Intuitive, Easy-to-Use, and Highly Customizable R Package to Generate Venn Diagram. Front. Genet..

[31] Carvalho A.S., Molina H., Matthiesen R. (2016). New Insights into Functional Regulation in MS-Based Drug Profiling. Sci. Rep..

[32] Matthiesen R., Prieto G., Amorim A., Aloria K., Fullaondo A., Carvalho A.S., Arizmendi J.M. (2012). SIR: Deterministic Protein Inference from Peptides Assigned to MS Data. J. Proteom..

[33] Ghuwalewala S., Ghatak D., Das P., Dey S., Sarkar S., Alam N., Panda C.K., Roychoudhury S. (2016). CD44 High CD24 Low Molecular Signature Determines the Cancer Stem Cell and EMT Phenotype in Oral Squamous Cell Carcinoma. Stem Cell Res..

[34] Zhang Z., Ma P., Jing Y., Yan Y., Cai M.C., Zhang M., Zhang S., Peng H., Ji Z.L., Di W. (2016). BET Bromodomain Inhibition as a Therapeutic Strategy in Ovarian Cancer by Downregulating Foxm1. Theranostics.

[35] Lim Y.C., Kang H.J., Kim Y.S., Choi E.C. (2012). All-Trans-Retinoic Acid Inhibits Growth of Head and Neck Cancer Stem Cells by Suppression of Wnt/β-Catenin Pathway. Eur. J. Cancer.

[36] Young M.J., Wu Y.H., Chiu W.T., Weng T.Y., Huang Y.F., Chou C.Y. (2014). All-Trans Retinoic Acid Downregulates ALDH1-Mediated Stemness and Inhibits Tumour Formation in Ovarian Cancer Cells. Carcinogenesis.

[37] Yao W., Wang L., Huang H., Li X., Wang P., Mi K., Cheng J., Liu H., Gu C., Huang L. (2020). All-Trans Retinoic Acid Reduces Cancer Stem Cell-like Cell-Mediated Resistance to Gefitinib in NSCLC Adenocarcinoma Cells. BMC Cancer.

[38] Qureshi-Baig K., Ullmann P., Rodriguez F., Frasquilho S., Nazarov P.V., Haan S., Letellier E. (2016). What Do We Learn from Spheroid Culture Systems? Insights from Tumorspheres Derived from Primary Colon Cancer Tissue. PLoS ONE.

[39] Ding D.-C., Liu H.-W., Chang Y.-H., Chu T.-Y. (2017). Expression of CD133 in Endometrial Cancer Cells and Its Implications. J. Cancer.

[40] Ginestier C., Hur M.H., Charafe-Jauffret E., Monville F., Dutcher J., Brown M., Jacquemier J., Viens P., Kleer C.G., Liu S. (2007). ALDH1 Is a Marker of Normal and Malignant Human Mammary Stem Cells and a Predictor of Poor Clinical Outcome. Cell Stem Cell.

[41] Mori Y., Yamawaki K., Ishiguro T., Yoshihara K., Ueda H., Sato A., Ohata H., Yoshida Y., Minamino T., Okamoto K. (2019). ALDH-Dependent Glycolytic Activation Mediates Stemness and Paclitaxel Resistance in Patient-Derived Spheroid Models of Uterine Endometrial Cancer. Stem Cell Rep..

[42] Laranjo M., Carvalho M.J., Costa T., Alves A., Oliveira R.C., Casalta-Lopes J., Cordeiro P., Botas F., Abrantes A.M., Paiva A. (2018). Mammospheres of Hormonal Receptor Positive Breast Cancer Diverge to Triple-Negative Phenotype. Breast.

[43] Chen G., Liu B., Yin S., Li S., Guo Y., Wang M., Wang K., Wan X. (2020). Hypoxia Induces an Endometrial Cancer Stem-like Cell Phenotype via HIF-Dependent Demethylation of SOX2 MRNA. Oncogenesis.

[44] Wang W., Zheng S., He H., Ge H., Saeed B. (2020). N,N-diethylaminobenzaldehyde Targets Aldehyde Dehydrogenase to Eradicate Human Pancreatic Cancer Cells. Exp. Ther. Med..

[45] Elbasateeny S.S., Salem A.A., Abdelsalam W.A., Salem R.A. (2016). Immunohistochemical Expression of Cancer Stem Cell Related Markers CD44 and CD133 in Endometrial Cancer. Pathol. Res. Pract..

[46] Kong F.-F., Li D., Yang H., Ma J., Pan X., Liu H.-X., Huo J.-N., Ma X.-X. (2017). Preliminary Identification of Endometrial Cancer Stem Cells in Vitro and in Vivo. Biochem. Biophys. Res. Commun..

[47] Silva I.A., Bai S., McLean K., Yang K., Griffith K., Thomas D., Ginestier C., Johnston C., Kueck A., Reynolds R.K. (2011). Aldehyde Dehydrogenase in Combination with CD133 Defines Angiogenic Ovarian Cancer Stem Cells That Portend Poor Patient Survival. Cancer Res..

[48] Liou G.Y. (2019). CD133 as a Regulator of Cancer Metastasis through the Cancer Stem Cells. Int. J. Biochem. Cell Biol..

[49] Janicek M.F., Angioli R., Unal A.D., Sevin B.U., Madrigal M., Estape R., Averette H.E. (1997). P53 Interference and Growth Inhibition in P53-Mutant and Overexpressing Endometrial Cancer Cell Lines. Gynecol. Oncol..

[50] Ghatak D., Das Ghosh D., Roychoudhury S. (2021). Cancer Stemness: P53 at the Wheel. Front. Oncol..

[51] Solomon H., Dinowitz N., Pateras I.S., Cooks T., Shetzer Y., Molchadsky A., Charni M., Rabani S., Koifman G., Tarcic O. (2018). Mutant P53 Gain of Function Underlies High Expression Levels of Colorectal Cancer Stem Cells Markers. Oncogene.

[52] Attarha S., Mints M., Andersson S., Souchelnytskyi S. (2011). Endometrial Cancer and Application of Proteomics. Exp. Oncol..

[53] Attarha S., Andersson S., Mints M., Souchelnytskyi S. (2013). Individualised Proteome Profiling of Human Endometrial Tumours Improves Detection of New Prognostic Markers. Br. J. Cancer.

[54] Pontén F., Jirström K., Uhlen M. (2008). The Human Protein Atlas—A Tool for Pathology. J. Pathol..

[55] Human Protein Atlas. https://www.proteinatlas.org/ENSG00000059573-ALDH18A1/pathology/endometrial+cancer.

[56] Wang L., Cybula M., Rostworowska M., Wang L., Mucha P., Bulicz M., Bieniasz M. (2022). Upregulation of Succinate Dehydrogenase (SDHA) Contributes to Enhanced Bioenergetics of Ovarian Cancer Cells and Higher Sensitivity to Anti-Metabolic Agent Shikonin. Cancers.

[57] Yoshida K., Kajiyama H., Inami E., Tamauchi S., Ikeda Y., Yoshikawa N., Nishino K., Utsumi F., Niimi K., Suzuki S. (2020). Clinical Significance of Ubiquitin-Associated Protein 2-like in Patients with Uterine Cervical Cancer. In Vivo.

[58] Lin S., Yan Z., Tang Q., Zhang S. (2021). Ubiquitin-Associated Protein 2 like (UBAP2L) Enhances Growth and Metastasis of Gastric Cancer Cells. Bioengineered.

[59] Li Q., Wang W., Hu Y.C., Yin T.T., He J. (2018). Knockdown of Ubiquitin Associated Protein 2-like (UBAP2L) Inhibits Growth and Metastasis of Hepatocellular Carcinoma. Med. Sci. Monit..

[60] Duffy M.J., O’Grady S., Tang M., Crown J. (2021). MYC as a Target for Cancer Treatment. Cancer Treat. Rev..

[61] Oshi M., Patel A., Le L., Tokumaru Y., Yan L., Matsuyama R., Endo I., Takabe K. (2021). G2M Checkpoint Pathway Alone Is Associated with Drug Response and Survival among Cell Proliferation-Related Pathways in Pancreatic Cancer. Am. J. Cancer Res..

[62] Li S., Shi B., Liu X., An H.X. (2020). Acetylation and Deacetylation of DNA Repair Proteins in Cancers. Front. Oncol..

[63] Zanoni M., Bravaccini S., Fabbri F., Arienti C. (2022). Emerging Roles of Aldehyde Dehydrogenase Isoforms in Anti-Cancer Therapy Resistance. Front. Med..

[64] Duan J.J., Cai J., Gao L., Yu S.C. (2023). ALDEFLUOR Activity, ALDH Isoforms, and Their Clinical Significance in Cancers. J. Enzyme Inhib. Med. Chem..

[65] Ibrahim A.I.M., Batlle E., Sneha S., Jiménez R., Pequerul R., Parés X., Rüngeler T., Jha V., Tuccinardi T., Sadiq M. (2022). Expansion of the 4-(Diethylamino)Benzaldehyde Scaffold to Explore the Impact on Aldehyde Dehydrogenase Activity and Antiproliferative Activity in Prostate Cancer. J. Med. Chem..

[66] Deutsch E.W., Bandeira N., Sharma V., Perez-Riverol Y., Carver J.J., Kundu D.J., García-Seisdedos D., Jarnuczak A.F., Hewapathirana s., Pullman B.S. (2020). The ProteomeXchange consortium in 2020: Enabling ‘big data’ approaches in proteomics. Nucleic Acids Res..

[67] Perez-Riverol Y., Csordas A., Bai J., Bernal-Llinares M., Hewapathirana S., Kundu D.J., Inuganti A., Griss J., Mayer G., Eisenacher M. (2019). The PRIDE database and related tools and resources in 2019: Improving support for quantification data. Nucleic Acids Res..

