# Supplementary material for: Influence of Aldehyde Dehydrogenase Inhibition on Stemness of Endometrial Cancer Stem Cells
Source: Cancers (Basel). 2024 May 27;16(11):2031. doi: 10.3390/cancers16112031 (PMC11171353; doi:10.3390/cancers16112031)

Supplementary Material

Figure S1 - Expression of ALDH 1/2 in RL95-2 cell line, RL95-2 CSC and RL95-2 G1. Samples derived from parallel experiments and gels/blots were processed in parallel.

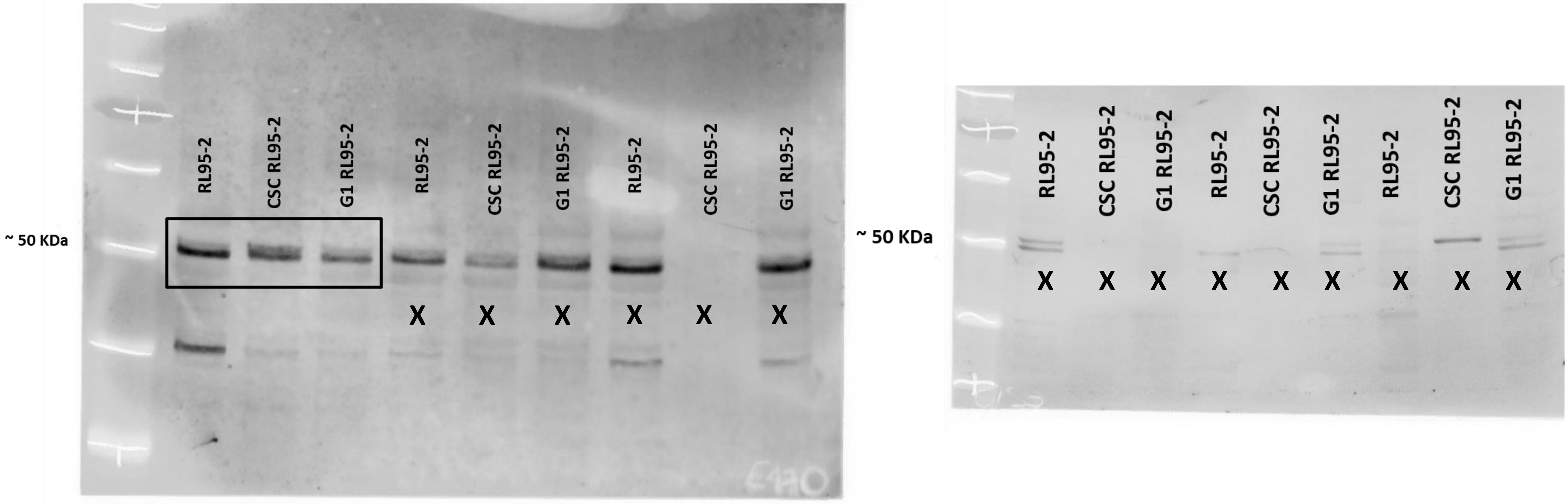

Supplementary Material

Figure S1 - Expression of Actin in RL95-2 cell line, RL95-2 CSC and RL95-2 G1. Samples derived from parallel experiments and gels/blots were processed in parallel.

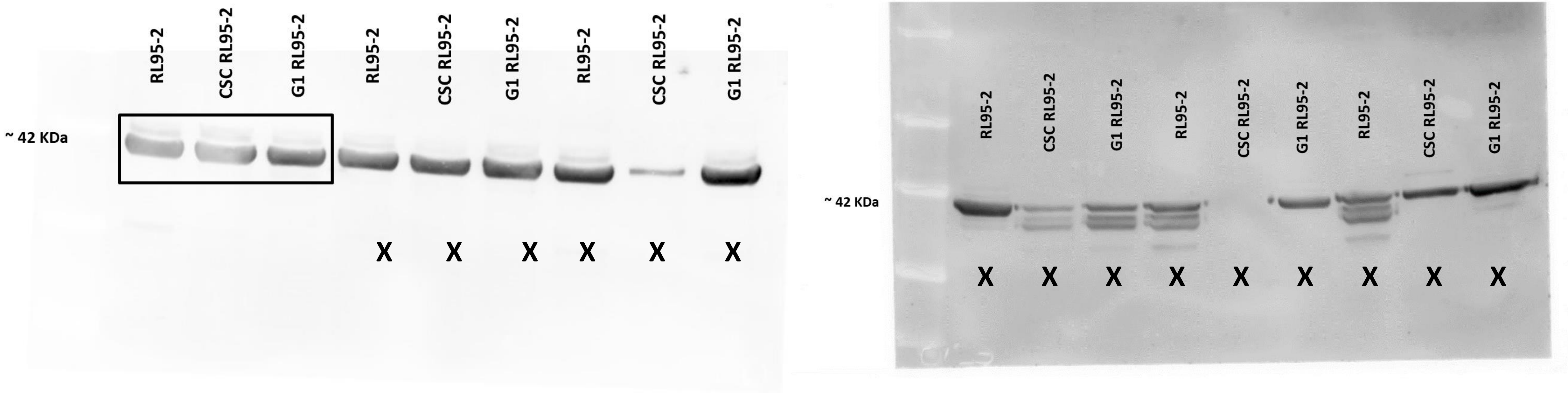

Supplementary Material

Figure S1 - Expression of P53 in RL95-2 cell line, RL95-2 CSC and RL95-2 G1

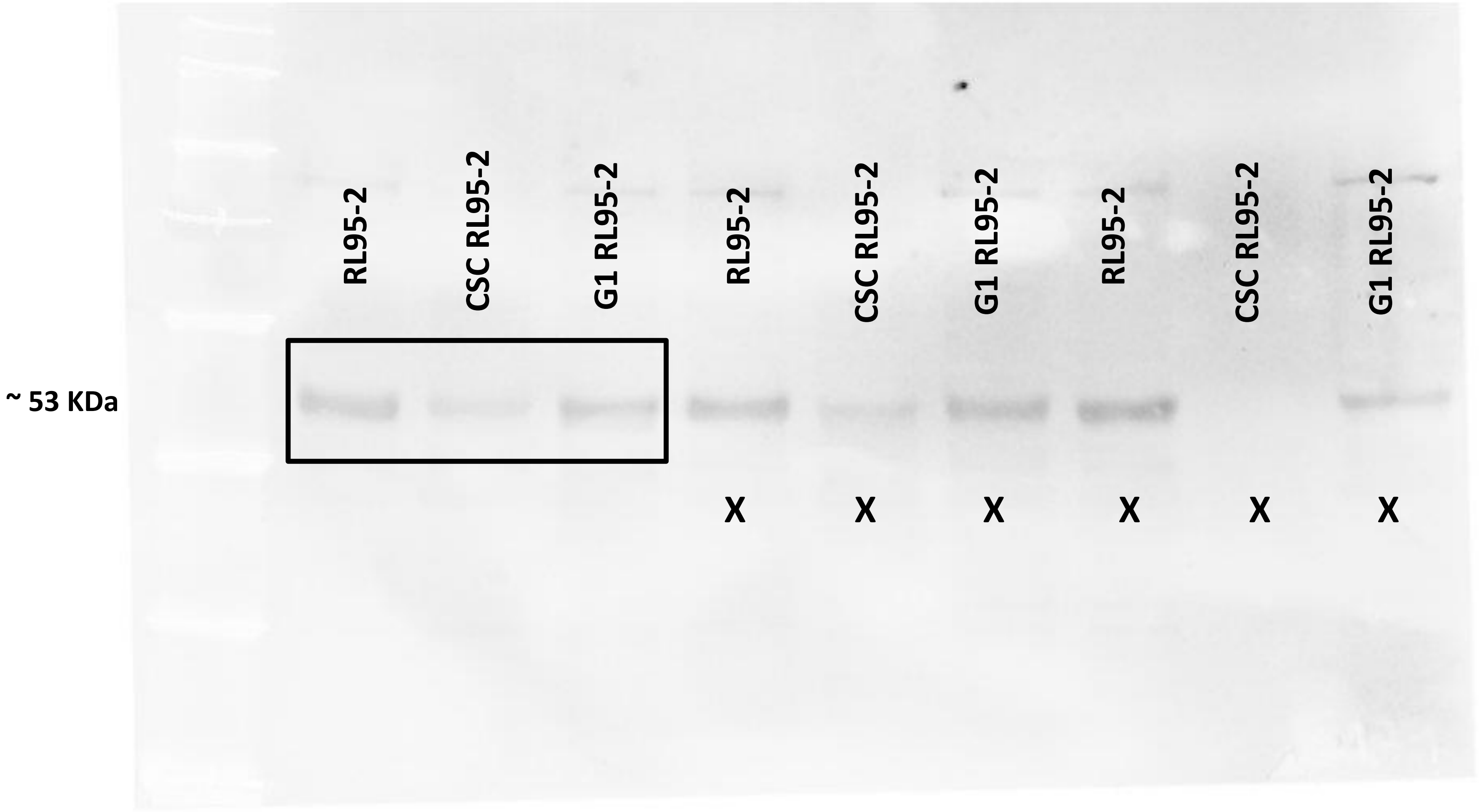

Supplementary Material

Figure S1 - Expression of Actin in RL95-2 cell line, RL95-2 CSC and RL95-2 G1

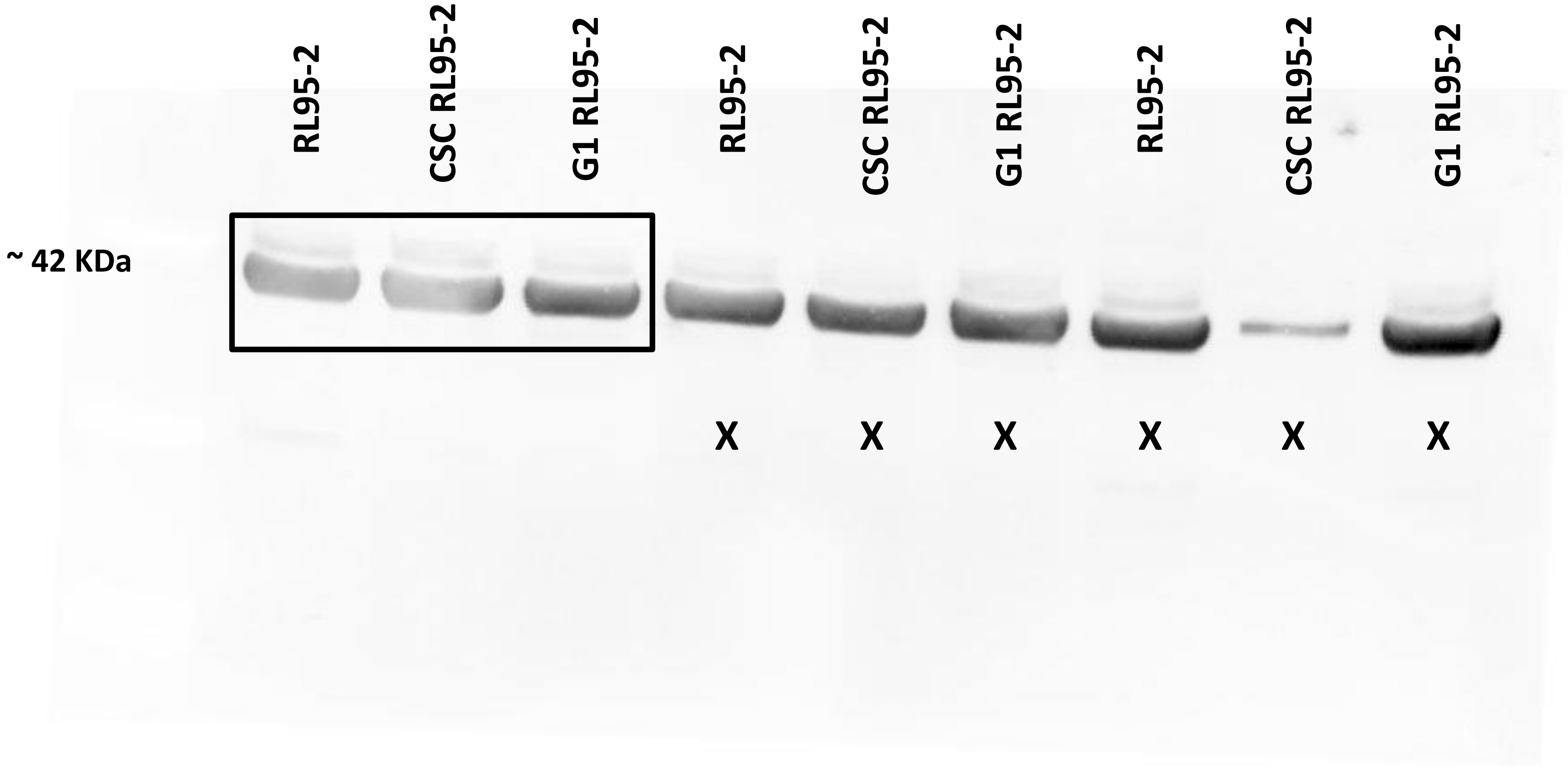

Supplementary Material

Figure S3 - Expression of ALDH in ECC-1 and RL95-2 cell lines under influence of ALDH inhibitors

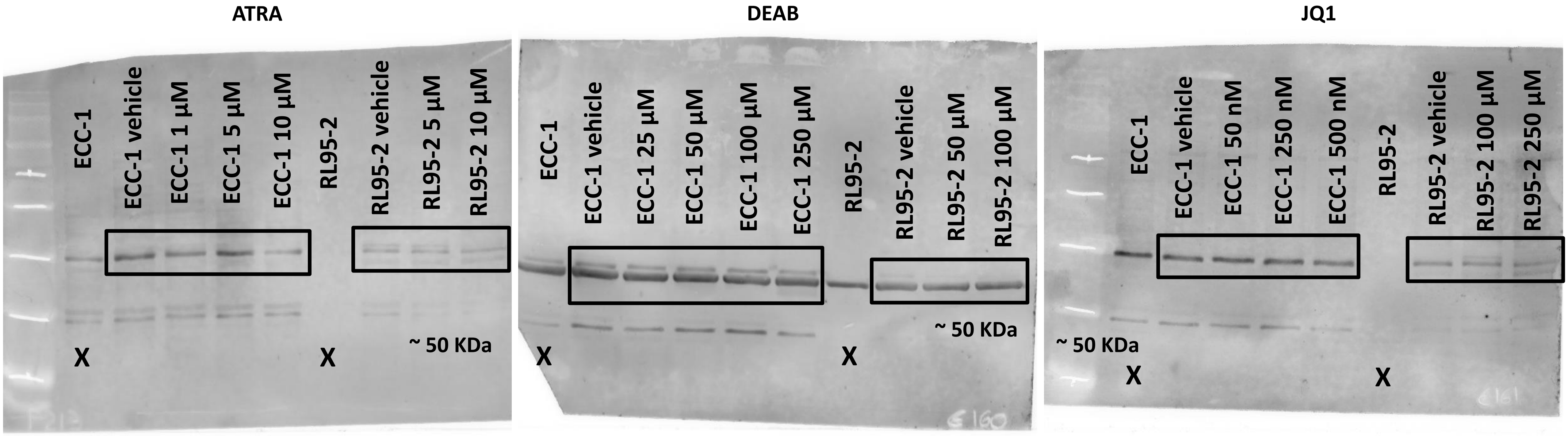

Supplementary Material

Figure S3 - Expression of Actin in ECC-1 and RL95-2 cell lines under influence of ALDH inhibitors

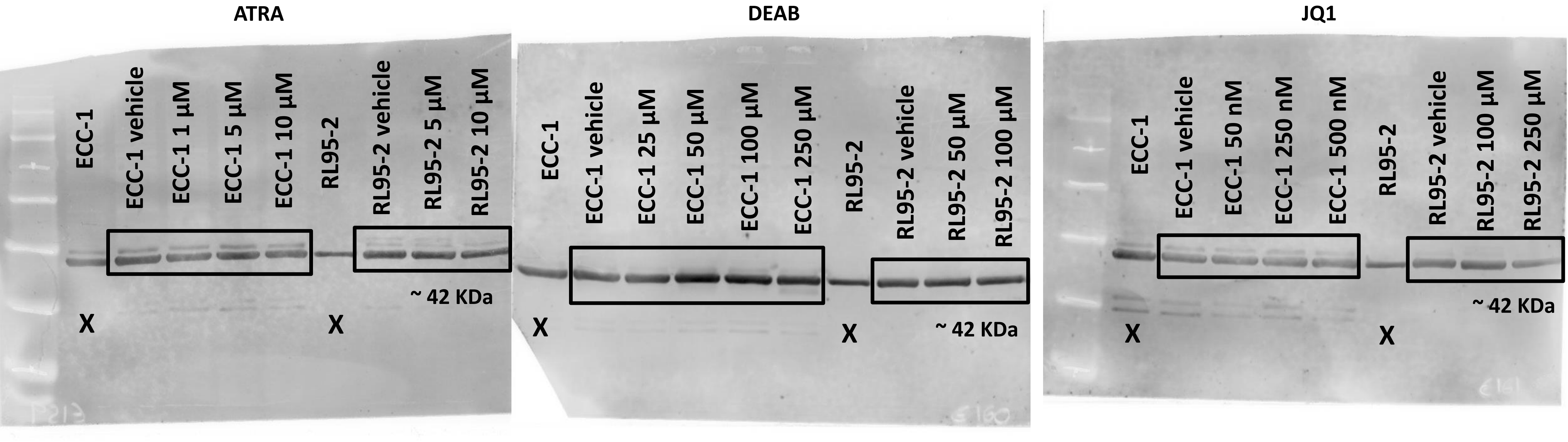

Article

Figure 1 - Expression of ALDH on ECC-1 CSC under influence of DEAB

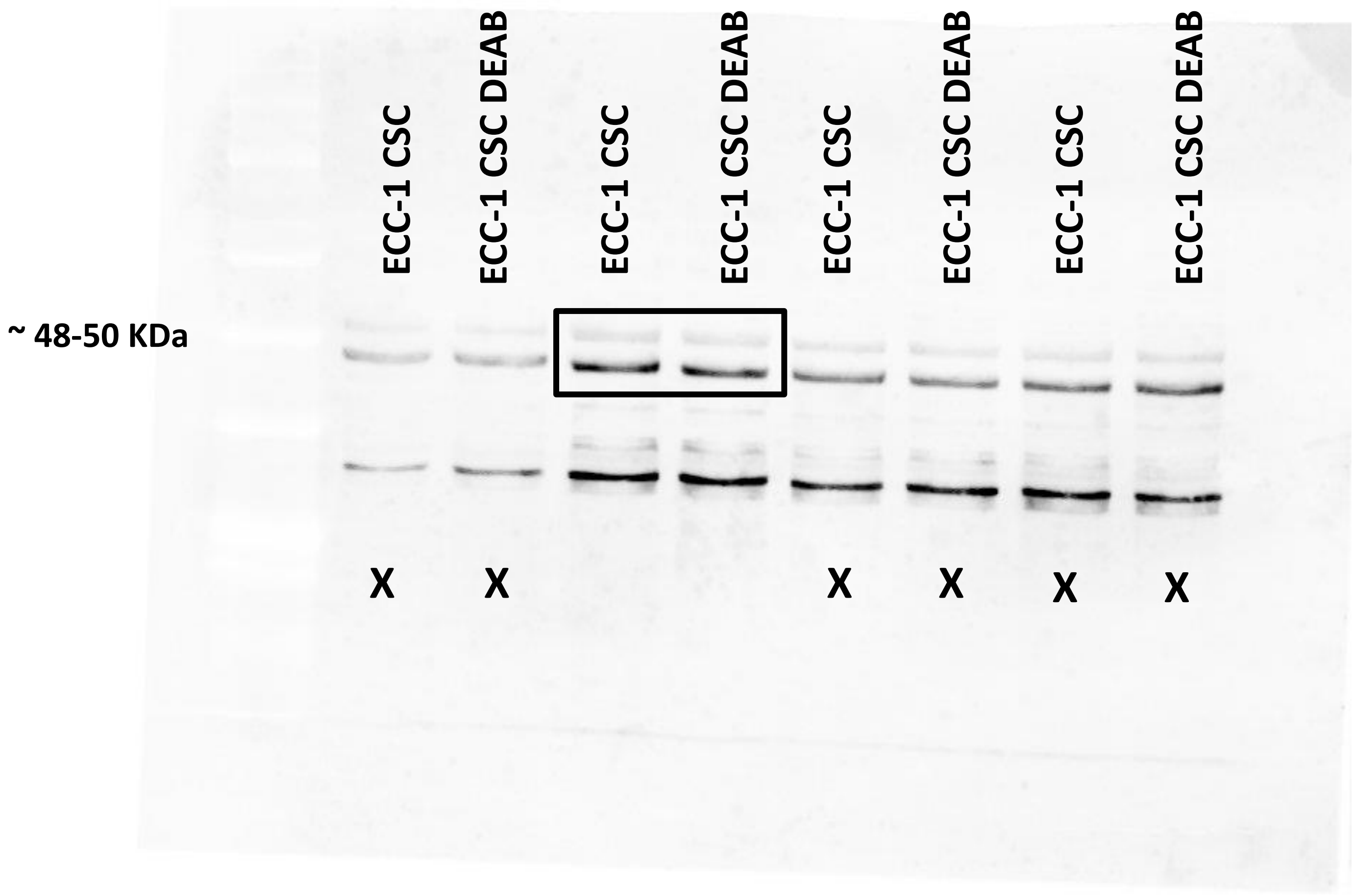

Article

Figure 1 - Expression of Actin on ECC-1 CSC under influence of DEAB

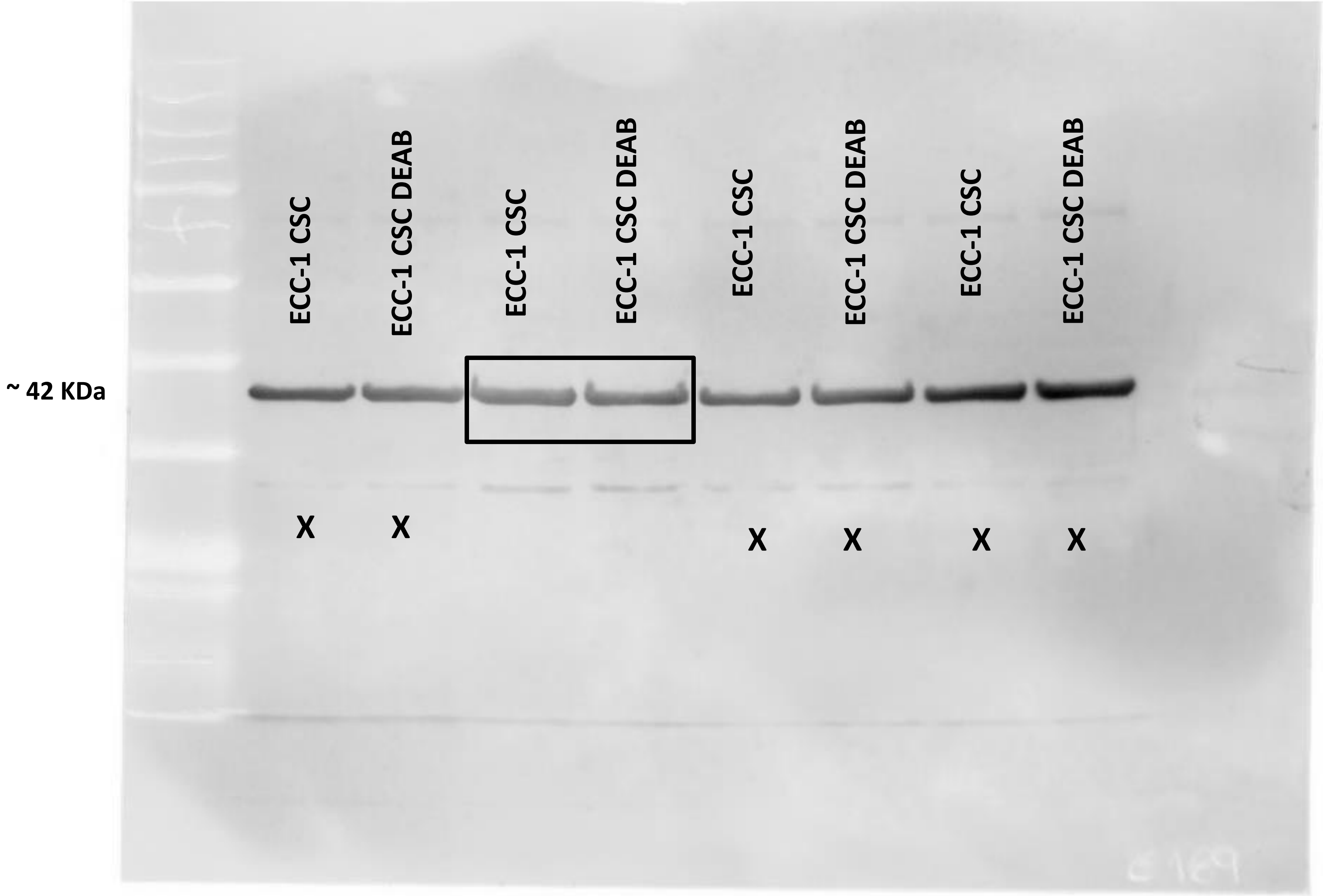

Article

Figure 1 - Expression of ALDH on RL95-2 CSC under influence of DEAB

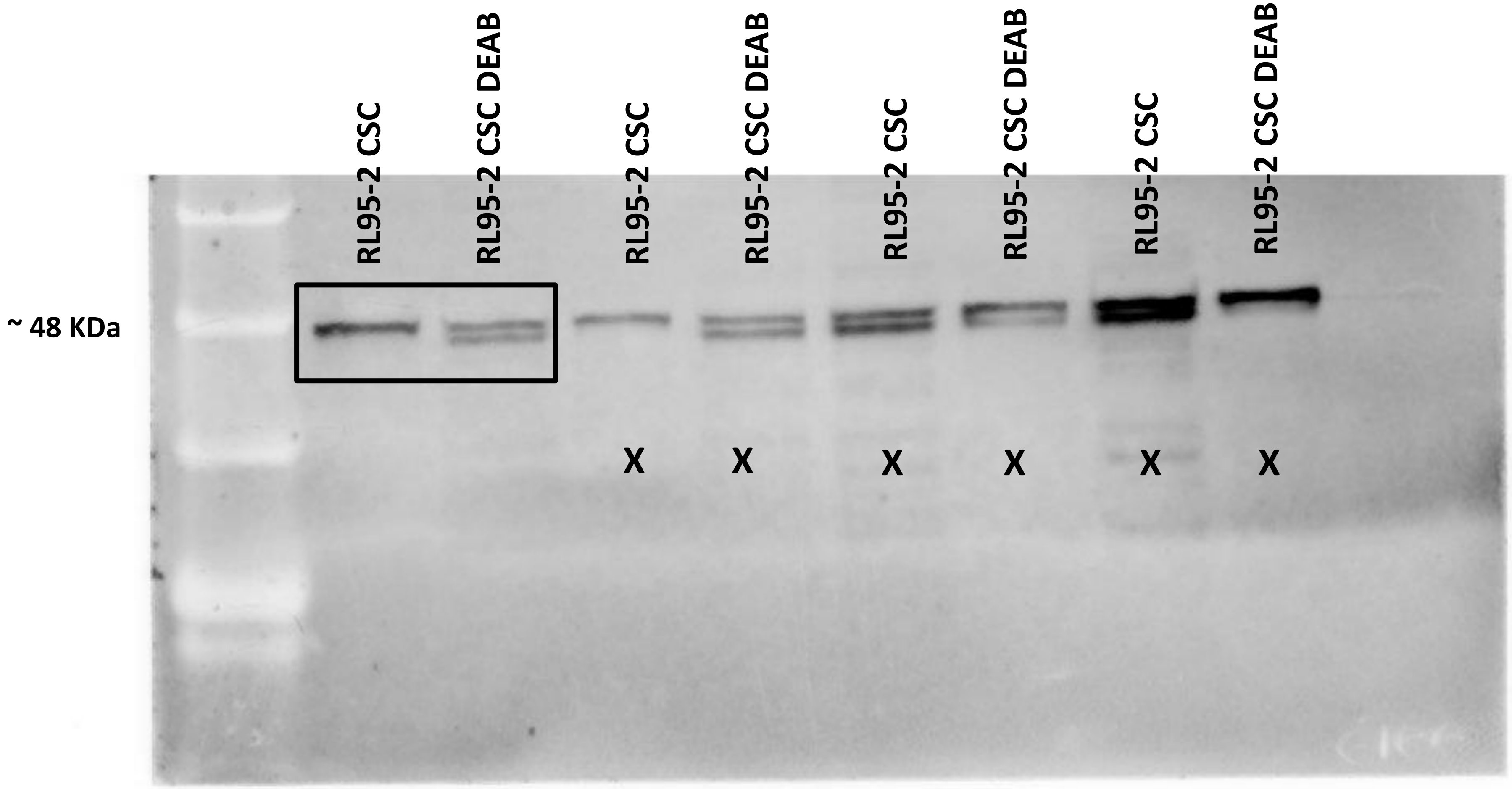

Article

Figure 1 - Expression of Actin on RL95-2 CSC under influence of DEAB

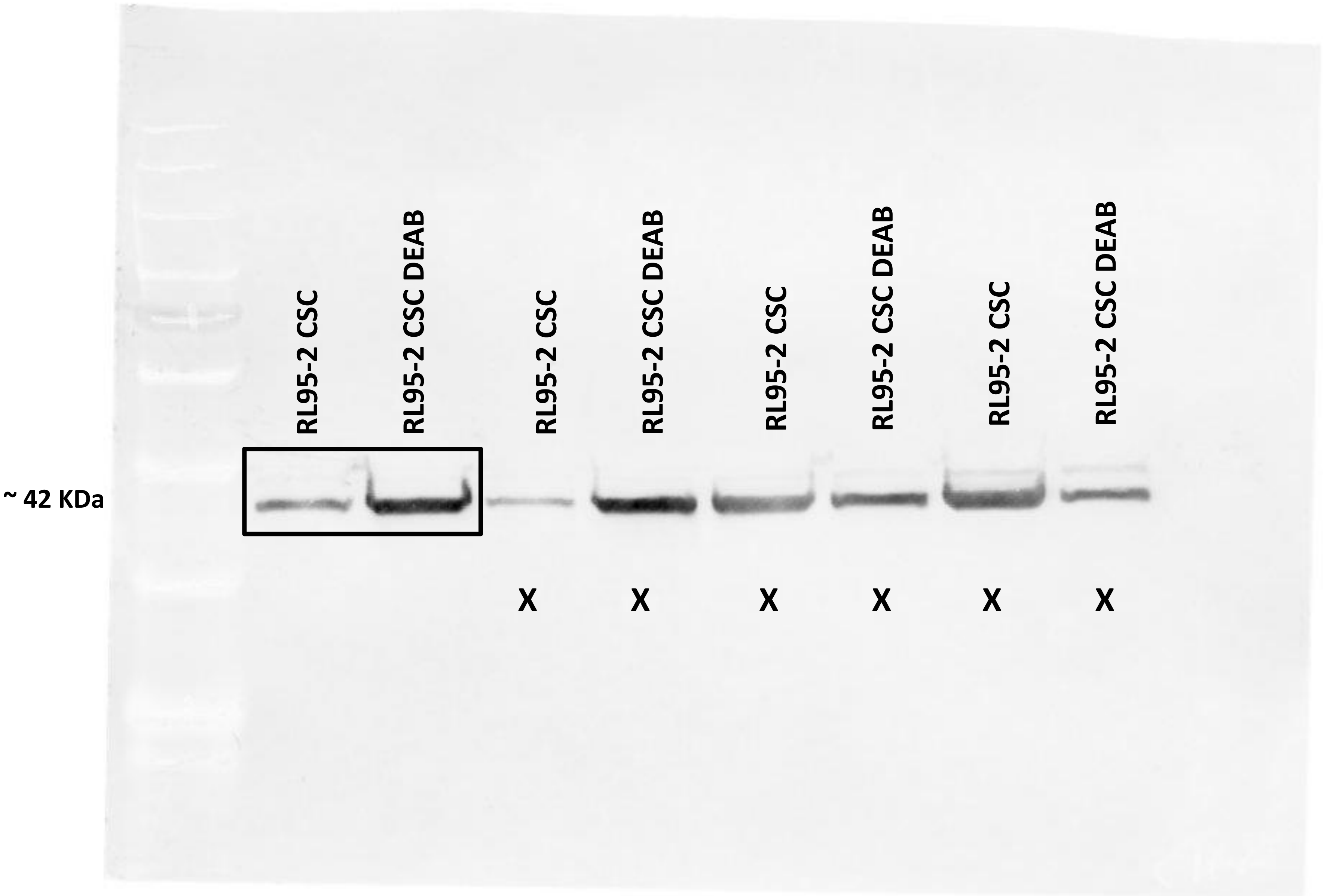

Article

Figure 4 - Expression of P53 on ECC-1 CSC under influence of DEAB

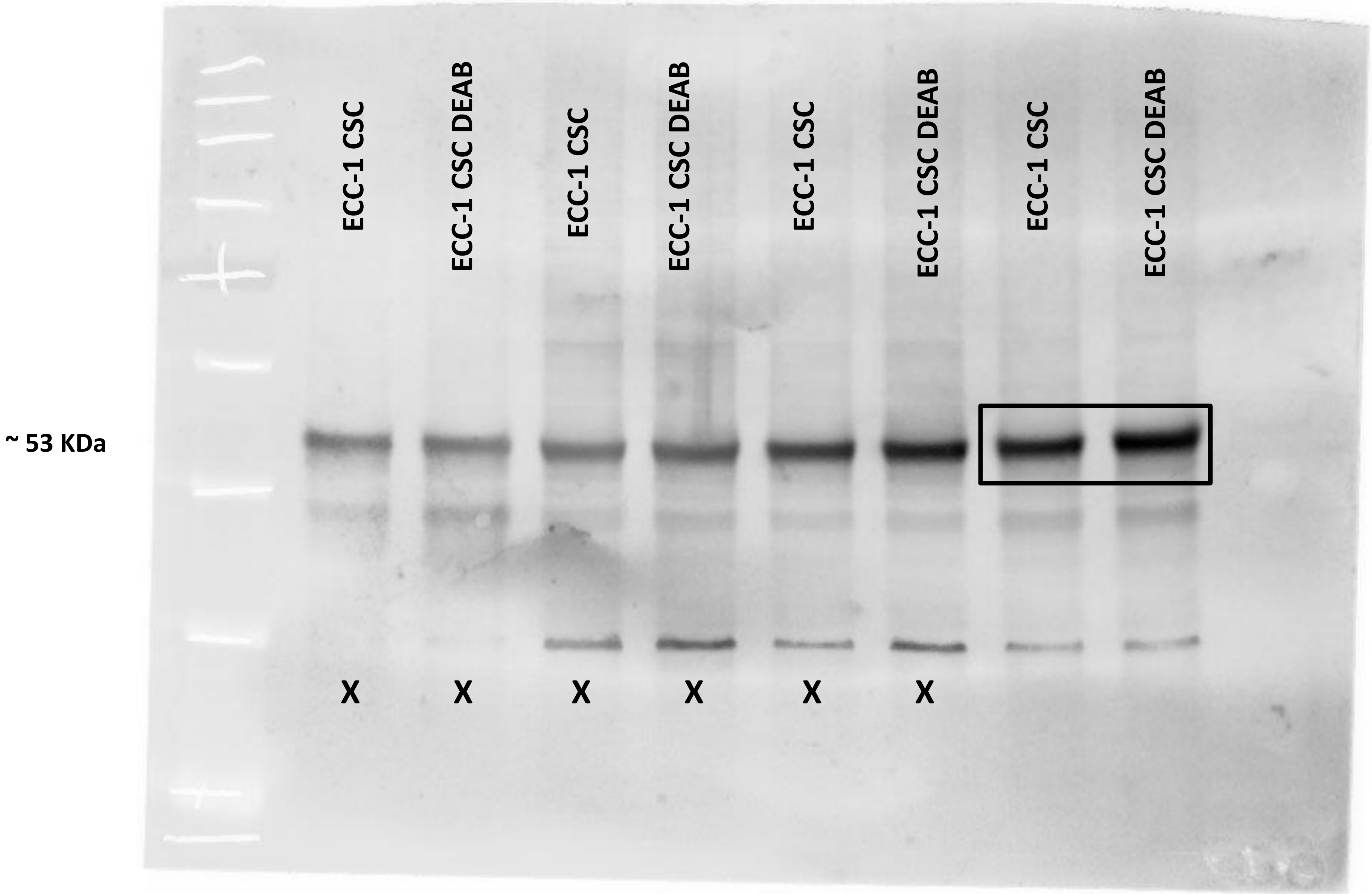

Article

Figure 4 - Expression of Actin on ECC-1 CSC under influence of DEAB

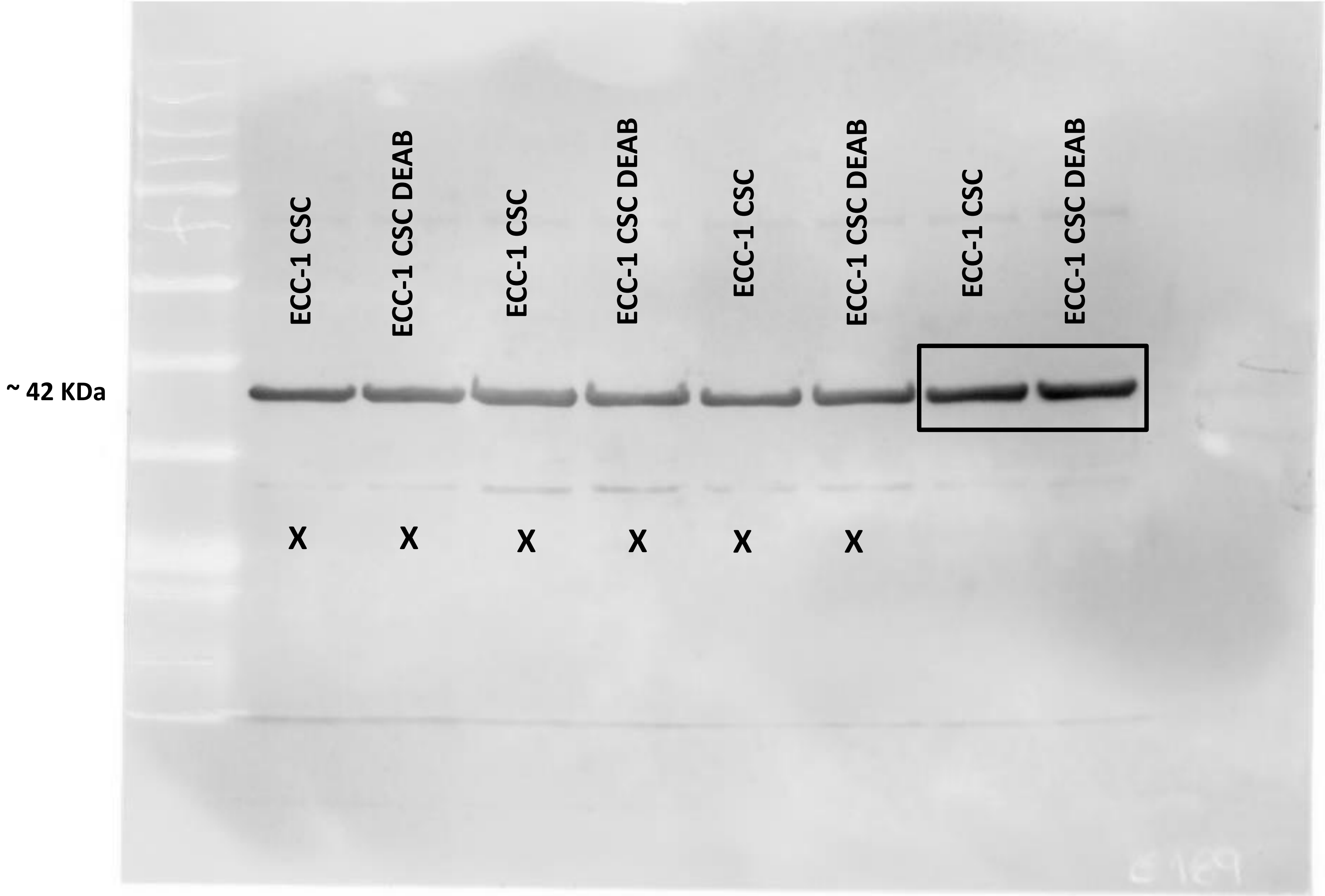

Article

Figure 4 - Expression of P53 on RL95-2 CSC under influence of DEAB

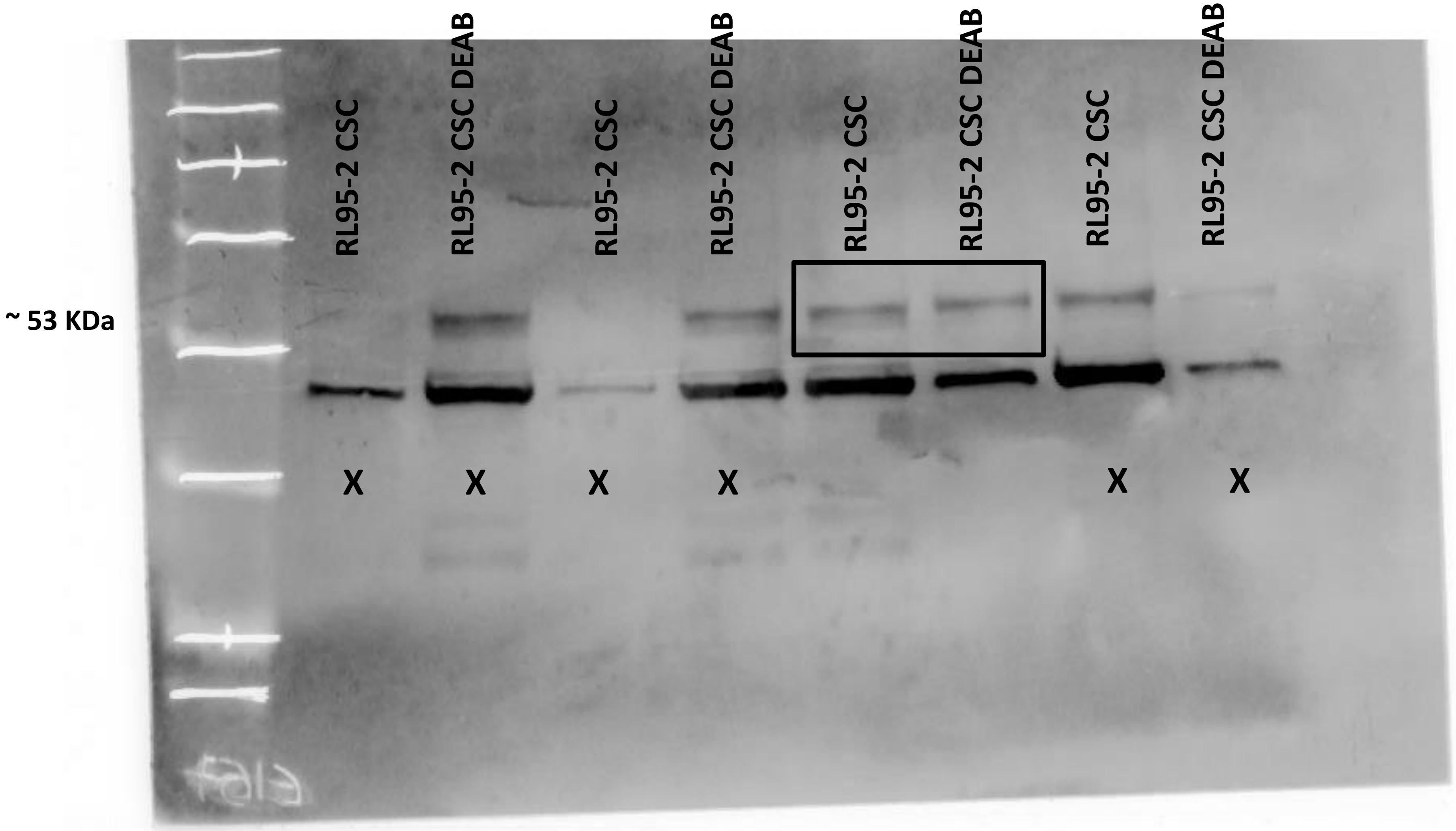

Article

Figure 4 - Expression of Actin on RL95-2 CSC under influence of DEAB

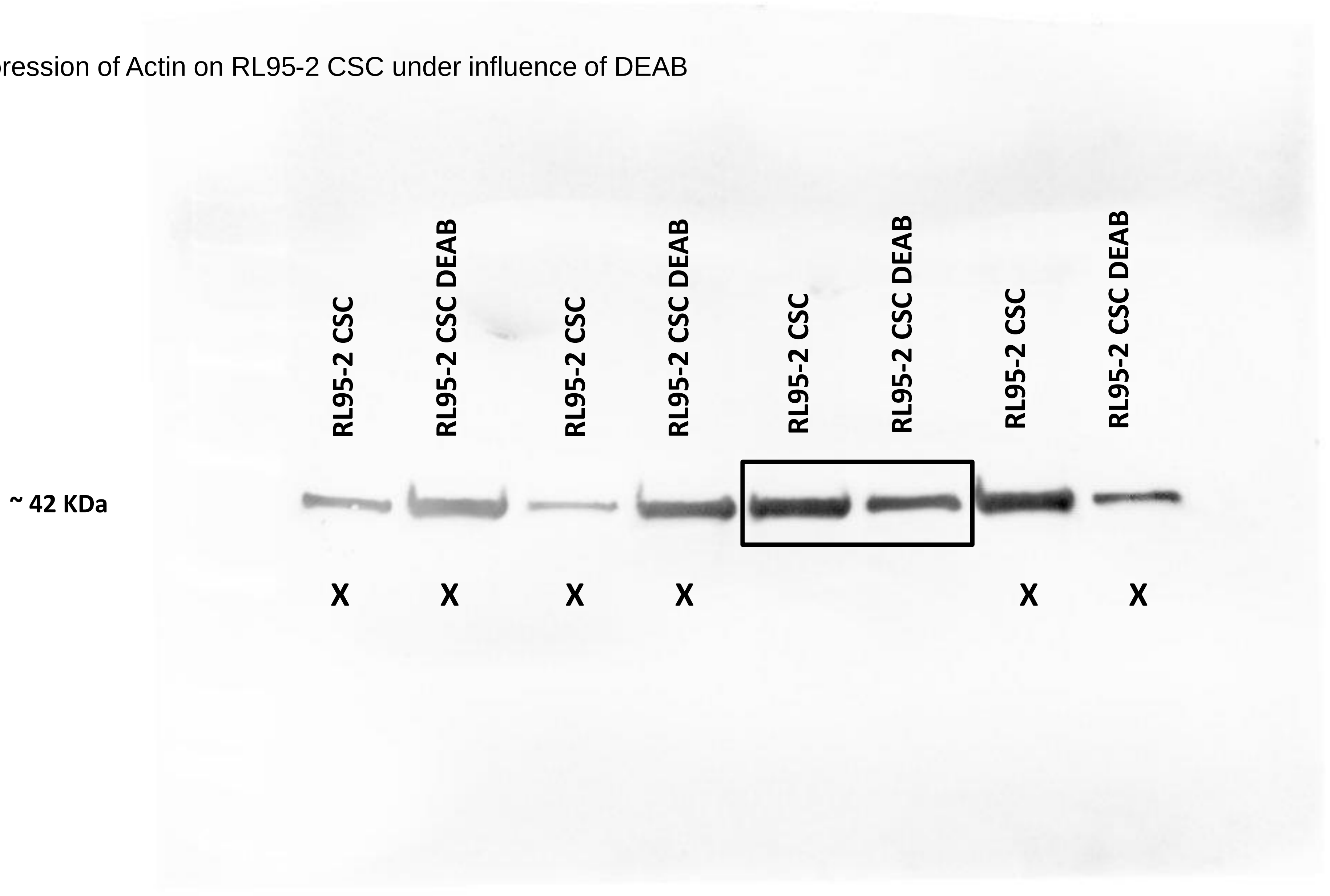

Supplement: Supplementary file 1 [file cancers-16-02031-s001.zip › File S1-Original western blots.pdf]
