# Supplementary material for: Influence of Aldehyde Dehydrogenase Inhibition on Stemness of Endometrial Cancer Stem Cells
Source: Cancers (Basel). 2024 May 27;16(11):2031. doi: 10.3390/cancers16112031 (PMC11171353; doi:10.3390/cancers16112031)
Supplement: Supplementary file 1 [file cancers-16-02031-s001.zip › supplementary figures.pdf]

cancers MDPI

Supplementary Material

Influence of Aldehyde Dehydrogenase Inhibition on Stemness of Endometrial Cancer Stem Cells

Beatriz Serambeque [1,2,*], Catarina Mestre [1,2], Gabriela Correia-Barros [1,2], Ricardo Teixo [1,2], Carlos Miguel Marto [1,2,3,4], Ana Cristina Gonçalves [2,4,5], Francisco Caramelo [2,4,6], Isabel Silva [7], Artur Paiva [2,7,8], Hans C. Beck [9], Ana Sofia Carvalho [10], Maria Filomena Botelho [1,2,3,4], Maria João Carvalho [1,2,4,11,12], Rune Matthiesen [10] and Mafalda Laranjo [1,2,4,*]

1 Univ Coimbra, Coimbra Institute for Clinical and Biomedical Research (iCBR) Area of Environment Genetics and Oncobiology (CIMAGO), Institute of Biophysics, Faculty of Medicine, 3000-548 Coimbra, Portugal; uc48103@uc.pt (C.M.); uc2019169084@student.uc.pt (G.C.-B.); uc2008114703@student.uc.pt (R.T.); cmiguel.marto@uc.pt (C.M.M.); mfbotelho@fmed.uc.pt (M.F.B.); mjcarvalho@fmed.uc.pt (M.J.C.)
2 Univ Coimbra, Center for Innovative Biomedicine and Biotechnology (CIBB), 3000-548 Coimbra, Portugal; acgoncalves@fmed.uc.pt (A.C.G.); fcaramelo@fmed.uc.pt (F.C.); artur.paiva@chuc.min-saude.pt (A.P.)
3 Univ Coimbra, Institute of Experimental Pathology, Faculty of Medicine, 3000-548 Coimbra, Portugal
4 Clinical Academic Centre of Coimbra (CACC), 3004-561 Coimbra, Portugal
5 Univ Coimbra, Coimbra Institute for Clinical and Biomedical Research (iCBR) Area of Environment Genetics and Oncobiology (CIMAGO), Laboratory of Oncobiology and Hematology (LOH) and University Clinics of Hematology and Oncology, Faculty of Medicine, 3000-548 Coimbra, Portugal
6 Univ Coimbra, Coimbra Institute for Clinical and Biomedical Research (iCBR) Area of Environment Genetics and Oncobiology (CIMAGO) and Laboratory of Biostatistics and Medical Informatics (LBIM), Faculty of Medicine, 3004-531 Coimbra, Portugal
7 Cytometry Operational Management Unit, Clinical Pathology Department, Unidade de Saúde Local de Coimbra, 3004-561 Coimbra, Portugal; 14546@ulscoimbra.min-saude.pt
8 Polytechnic Institute of Coimbra, Coimbra Health School, Laboratory Biomedical Sciences, 3045-043 Coimbra, Portugal
9 Department of Clinical Biochemistry, Odense University Hospital, 5000 Odense, Denmark; hcbeck@health.sdu.dk
10 iNOVA4Health, NOVA Medical School (NMS), Faculdade de Ciências Médicas (FCM), Universidade Nova de Lisboa, 1150-082 Lisboa, Portugal; ana.carvalho@nms.unl.pt (A.S.C.); rune.matthiesen@nms.unl.pt (R.M.)
11 Univ Coimbra, Universitary Clinic of Gynecology, Faculty of Medicine, 3004-561 Coimbra, Portugal
12 Gynecology Service, Department of Gynecology, Obstetrics, Reproduction and Neonatology, Unidade Local de Saúde de Coimbra, 3004-561 Coimbra, Portugal
* Correspondence: beatriz.serambeque@student.uc.pt (B.S.); mafaldalaranjo@fmed.uc.pt (M.L.)

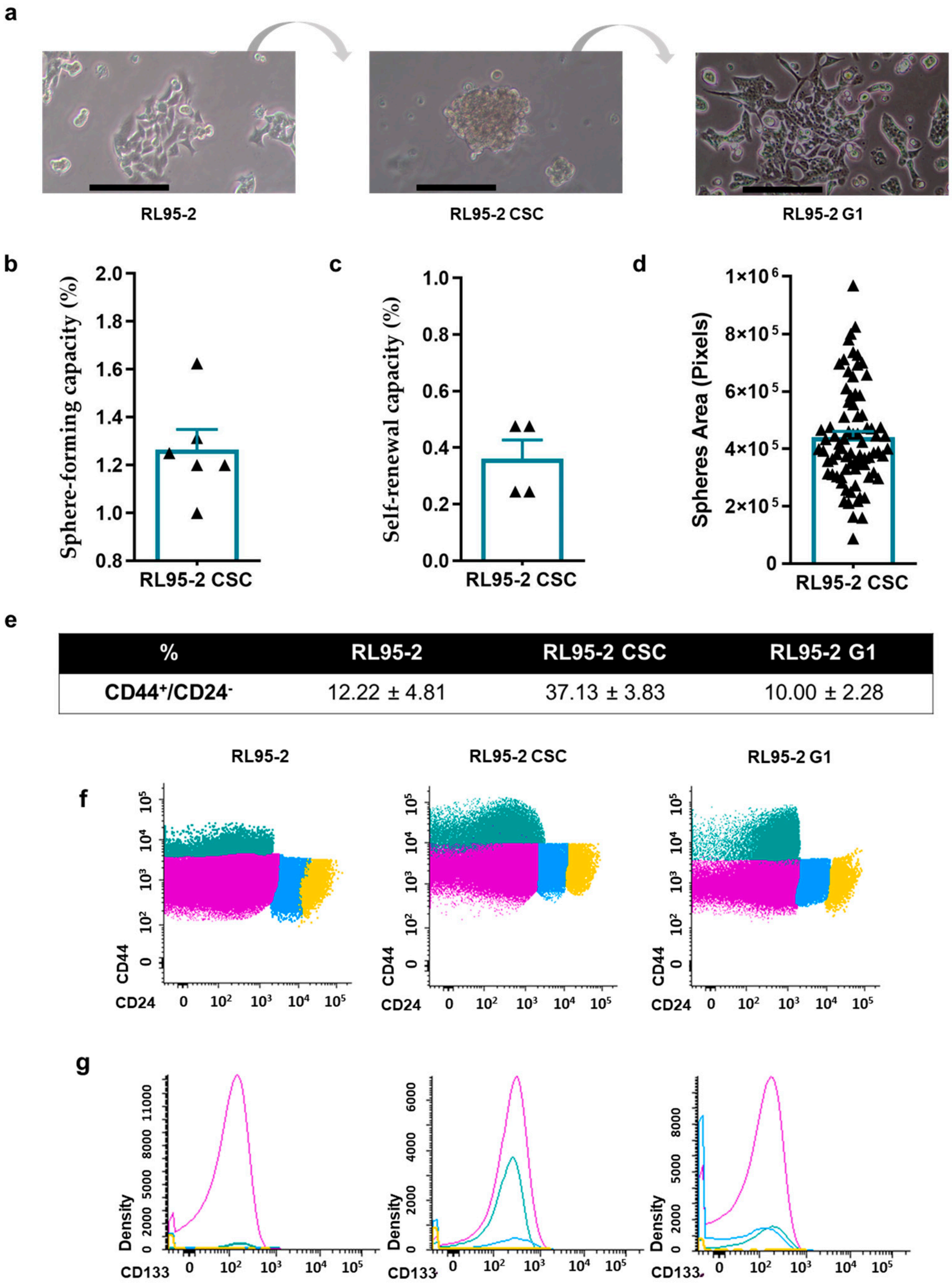
a
RL95-2
RL95-2 CSC
RL95-2 G1
b
Sphere-forming capacity (%)
RL95-2 CSC
c
Self-renewal capacity (%)
RL95-2 CSC
d
Spheres Area (Pixels)
RL95-2 CSC
e
% RL95-2 RL95-2 CSC RL95-2 G1
CD44+/CD24- 12.22 ± 4.81 37.13 ± 3.83 10.00 ± 2.28
f
RL95-2
RL95-2 CSC
RL95-2 G1
CD44
CD24
g
Density
CD133

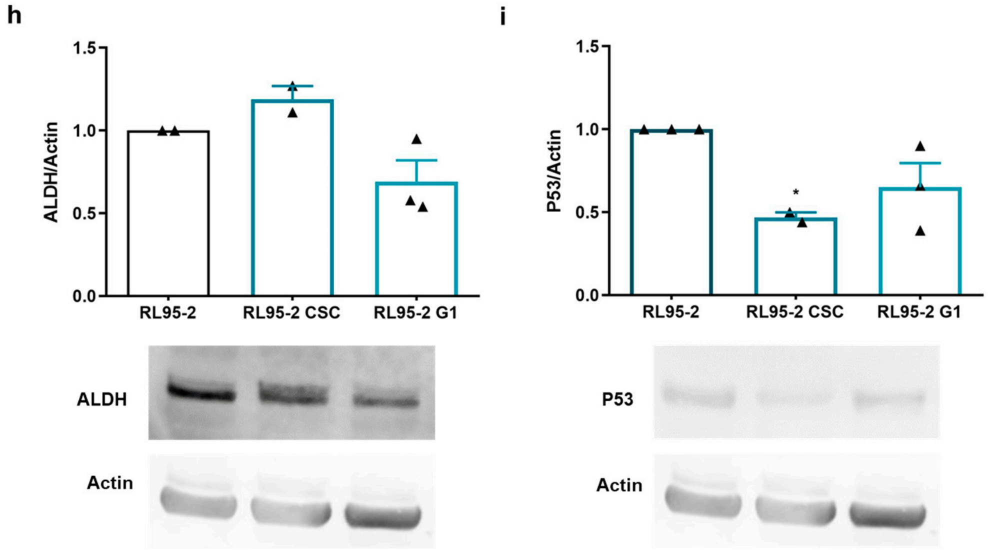

Figure S1 – Characterisation of endometrial cancer RL95-2 CSCs. **(a)** RL95-2 cells, CSC enriched colonies (named RL95-2 CSCs), and RL95-2 CSC derived adherent populations (named RL95-2 G1). Images were obtained at 100x magnification. Scale bar represents 100 µm. **(b)** Sphere-forming capacity of the RL95-2 cells. The results correspond to the mean and standard error of six independent experiments. **(c)** Self-renewal capacity of RL95-2 CSCs. The results correspond to the mean and standard error of two independent experiments in duplicate. **(d)** Sphere projection area of RL95-2 CSCs. The results correspond to the average of pixels and the standard error of 78 images obtained through three independent experiments. **(e)** Percentage of cells presenting the $CD44^+/CD24^-$ phenotype. The values express the mean and standard error of at least two independent experiments. **(f)** Representative plots of the CD44/CD24 expression. The green, pink, blue, and yellow correspond to the $CD44^+/CD24^-$, $CD44^{weak}/CD24^-$, $CD44^{weak}/CD24^{\pm}$, and $CD44^-/CD24^+$ populations, respectively. **(g)** Representative histograms of CD133 expression in the $CD44^+/CD24$, $CD44^{weak}/CD24^-$, $CD44^{weak}/CD24^{\pm}$, and $CD44^-/CD24^+$ populations identified in RL95-2, RL95-2 CSCs, and RL95-2 G1 (same colour code). **(h)** Expression of ALDH1/2. The values correspond to the ratio between the fluorescence intensities of ALDH and β-actin (ALDH/control actin ratio equal to 1). The results correspond to the mean and standard error of at least two independent experiments. **(i)** Expression of P53. The values correspond to the ratio between the fluorescence intensities of P53 and β-actin (P53/control actin ratio equal to 1). The results correspond to the mean and standard error of three independent experiments. Statistical significance is represented with * for $p<0.050$. # represents absence of expression. Sections (a), (f), and (g) of this figure is adapted from Laranjo, M., Carvalho, M. J., Serambeque, B., Alves, A., Marto, C. M., Silva, I., Paiva, A., Botelho, M. F. Obtaining Cancer Stem Cell Spheres from Gynecological and Breast Cancer Tumors. J. Vis. Exp. (157), e60022, doi:10.3791/60022 (2020), with authorisation from the publisher.

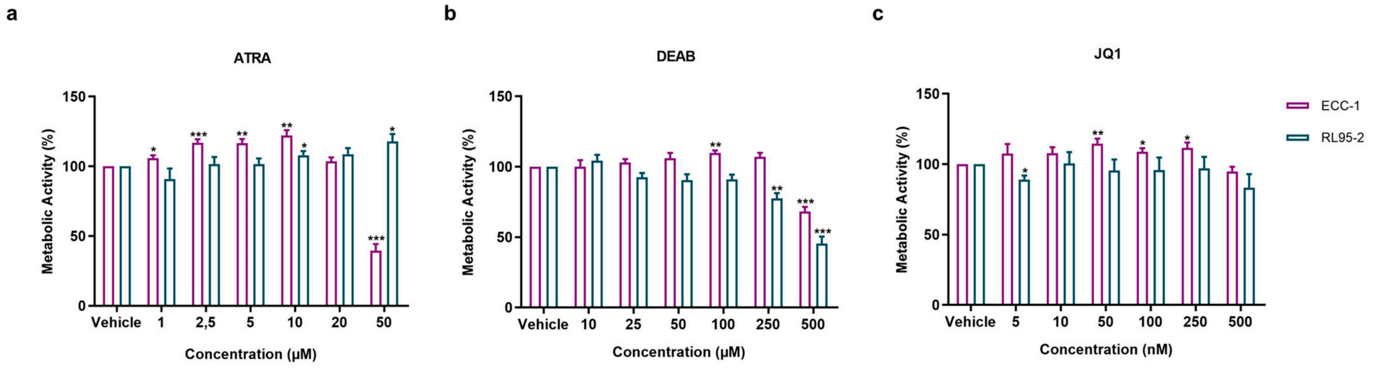

Figure S2 – Evaluation of the metabolic activity of human endometrial cancer cell lines, ECC-1 and RL95-2, after 48 hours of incubation with ALDH inhibitors **(a)** ATRA, **(b)** DEAB, and **(c)** JQ1. The results correspond to the mean and standard error of two independent experiments. Statistical significance is represented with * for $p<0.050$, ** for $p<0.010$ and *** for $p<0.001$.

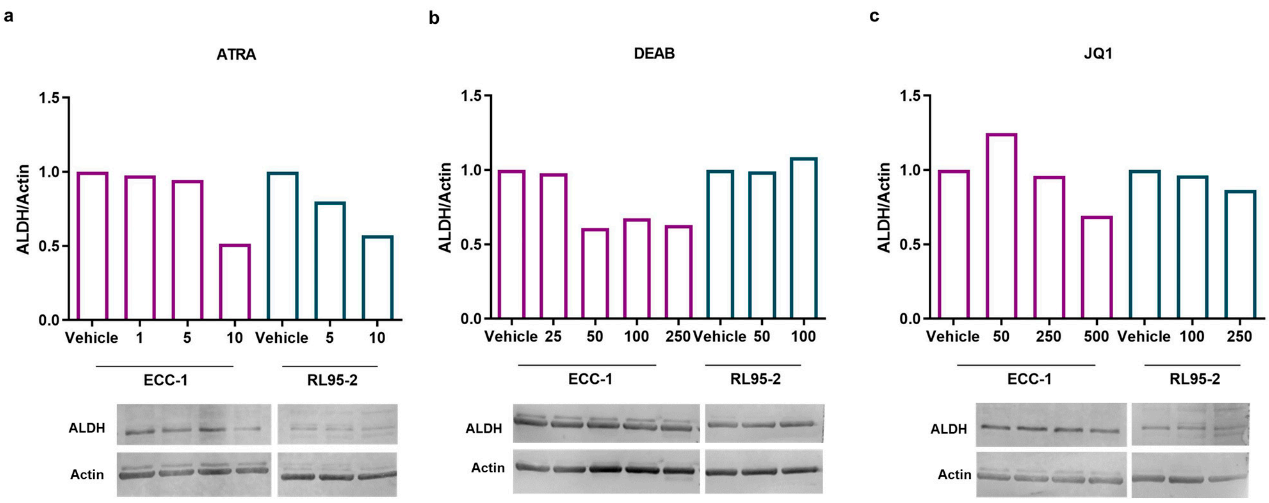

Figure S3 – Expression of ALDH in ECC-1 and RL95-2 cells treated with ATRA, DEAB, and JQ1. The values correspond to the ratio between the fluorescence intensities of ALDH and β-actin (ALDH/control actin ratio equal to 1). For the ALDH inhibition studies, endometrial cancer cell lines were incubated with ATRA, DEAB, and JQ1 for 48 hours. The results presented correspond to one experiment.
